# Hypoxia drives progression of multiple sclerosis by enhancing the inflammasome activation in macrophages with *Porphyromonas gingivalis* infection

**DOI:** 10.1038/s41420-025-02548-z

**Published:** 2025-06-10

**Authors:** Tokuju Okano, Hiroshi Ashida, Masayuki Tsukasaki, Tamako Iida, Masahiro Yamamoto, Hiroshi Takayanagi, Takeharu Sakamoto, Toshihiko Suzuki

**Affiliations:** 1 Department of Bacterial Pathogenesis, Infection and Host Response, Graduate School of Medical and Dental Sciences, Institute of Science Tokyo, Tokyo, Japan; 2 Department of Osteoimmunology, Graduate School of Medicine and Faculty of Medicine, The University of Tokyo, Tokyo, Japan; 3 Department of Biochemistry, School of Dentistry, Showa Medical University, Tokyo, Japan; 4 Department of Immunoparasitology, Research Institute for Microbial Diseases, Osaka University, Osaka, Japan; 5 Department of Immunology, Graduate School of Medicine and Faculty of Medicine, The University of Tokyo, Tokyo, Japan; 6 Department of Cancer Biology, Institute of Biomedical Science, Kansai Medical University, Hirakata, Japan

**Keywords:** Autoimmunity, Experimental models of disease, Bacterial infection

## Abstract

*Porphyromonas gingivalis* (*Pg*), a gram-negative anaerobic bacterium, is a leading pathogen causing periodontitis. It secretes several virulence factors, including gingipains, lipopolysaccharides (LPS), and outer membrane vesicles (OMVs), which can trigger the release of inflammatory cytokines such as interleukin (IL)-1β, tumor necrosis factor alpha (TNFα), and IL-6 through inflammasome activation and Toll-like receptor (TLR) signaling. We demonstrated that *Pg* infection under hypoxic conditions enhances NLRP3 inflammasome activation in macrophages. Additionally, we observed that toll-interleukin-1 receptor domain-containing adaptor-inducing interferon-β (TRIF)-mediated hypoxia-inducible factor 1 alpha (HIF-1α) regulation exacerbates inflammasome activation under hypoxia. Notably, HIF-1α deficiency in myeloid cells reversed neurological symptoms and reduced IL-1β and IL-17 production in a mouse model of multiple sclerosis with *Pg* infection. Our findings indicated that hypoxia modulates inflammasome activation in response to periodontitis-related bacterial infections, contributing to the progression of autoimmune diseases.

## Introduction

Periodontitis is a common chronic inflammatory disease linked to several autoimmune conditions, including rheumatoid arthritis, multiple sclerosis, and ulcerative colitis [1–4]. A bacterial group known as the “red complex,” which includes *Porphyromonas gingivalis*, *Treponema denticola*, and *Tannerella forsythia*, is recognized as a major contributor to periodontitis [5]. This complex regulates oral flora and inflammatory signaling pathways in both innate and adaptive immunity, thereby promoting the progression of the disease. Among the red complex bacteria, *Porphyromonas gingivalis*, a gram-negative anaerobe, is the most common cause of periodontitis [6–9]. It secretes several virulence factors, including gingipains, lipopolysaccharide (LPS), and outer membrane vesicles (OMVs), which activate the inflammasome and Toll-like receptors (TLRs), leading to the production of inflammatory cytokines such as interleukin (IL)-1β, tumor necrosis factor alpha (TNFα), and IL-6 [10–12]. The resulting host inflammatory response exacerbates tissue destruction in periodontitis, contributing to connective tissue and bone loss and is a leading cause of adult tooth loss.

Inflammasomes, composed of Nod-like receptors (NLRs), the adapter protein known as apoptosis-associated speck-like protein containing a caspase recruitment domain (ASC), and procaspase-1, are multi-protein complexes that detect damage-associated molecular patterns and pathogen-associated molecular patterns. Upon activation, they form heterodimeric structures known as ASC specks, which activate caspase-1 [13]. This, in turn, triggers the secretion of IL-1β and IL-18 and induces pyroptosis, a form of programmed cell death that plays critical roles in diseases such as type 2 diabetes, atherosclerosis, Parkinson’s disease, rheumatoid arthritis, and multiple sclerosis [14–18]. Previous studies using animal models have demonstrated that *Pg* activates inflammasomes in gingival fibroblasts and innate immune cells, exacerbating periodontitis [19–21]. Our study indicated that *Pg* induces inflammasome activation in a gingipain-independent manner, with gingipains contributing to cytokine degradation within cells [20]. However, the exact mechanisms by which *Pg* activates inflammasomes and contributes to periodontitis severity remain unclear.

In infection-related human tissues (e.g., colon, periodontal pockets), hypoxic conditions can influence immune responses. One major response to hypoxia is the activation of hypoxia-inducible factor-1 (HIF-1), a heterodimer composed of the hypoxia-responsive subunit HIF-1α and the constitutively expressed subunit HIF-1β. Under low oxygen levels, HIF-1 binds to hypoxia-response elements (HREs), activating genes such as glucose transporter 1 [22–25]. Some studies suggest that HIF-1α-regulated HREs are involved in the progression or suppression of infectious diseases, though the precise mechanisms and physiological functions remain poorly understood [26–28].

Multiple sclerosis (MS) is the most common chronic inflammatory disease of the central nervous system (CNS), characterized by demyelination and neurodegeneration. Numerous studies have highlighted the critical roles of both innate and adaptive immune cells, including macrophages, microglia, T cells, and B cells, in the development and progression of MS. In the experimental autoimmune encephalomyelitis (EAE) mouse model of MS, the expansion of pathogenic CD4+ T cells induces disease onset [29, 30]. Macrophages from the blood migrate to MS lesions, where they clear myelin debris and inflammatory by-products [31]. Similarly, microglia, the resident phagocytic cells of the CNS, are abundant in MS lesions, as seen in the white matter of both autopsy specimens and the EAE model [32]. Additionally, clinical and basic studies have established that inflammasome activation is a key molecular mechanism in the pathogenesis of MS and EAE [33].

In this study, we demonstrated that *Pg* infection under hypoxic conditions induces enhancement of inflammasome activation in macrophages via the TLR4-TRIF-MyD88-HIF-1α axis. We also discovered that oral infection with *Pg* drives IL-17 production and inflammasome activation in the spleen, exacerbating EAE in mice through a HIF-1α-dependent pathway.

## Results

### NLRP3 inflammasome activation and cytotoxicity induced by *Pg* infection or its secreted proteins are upregulated under hypoxic conditions

**Fig. 1:**
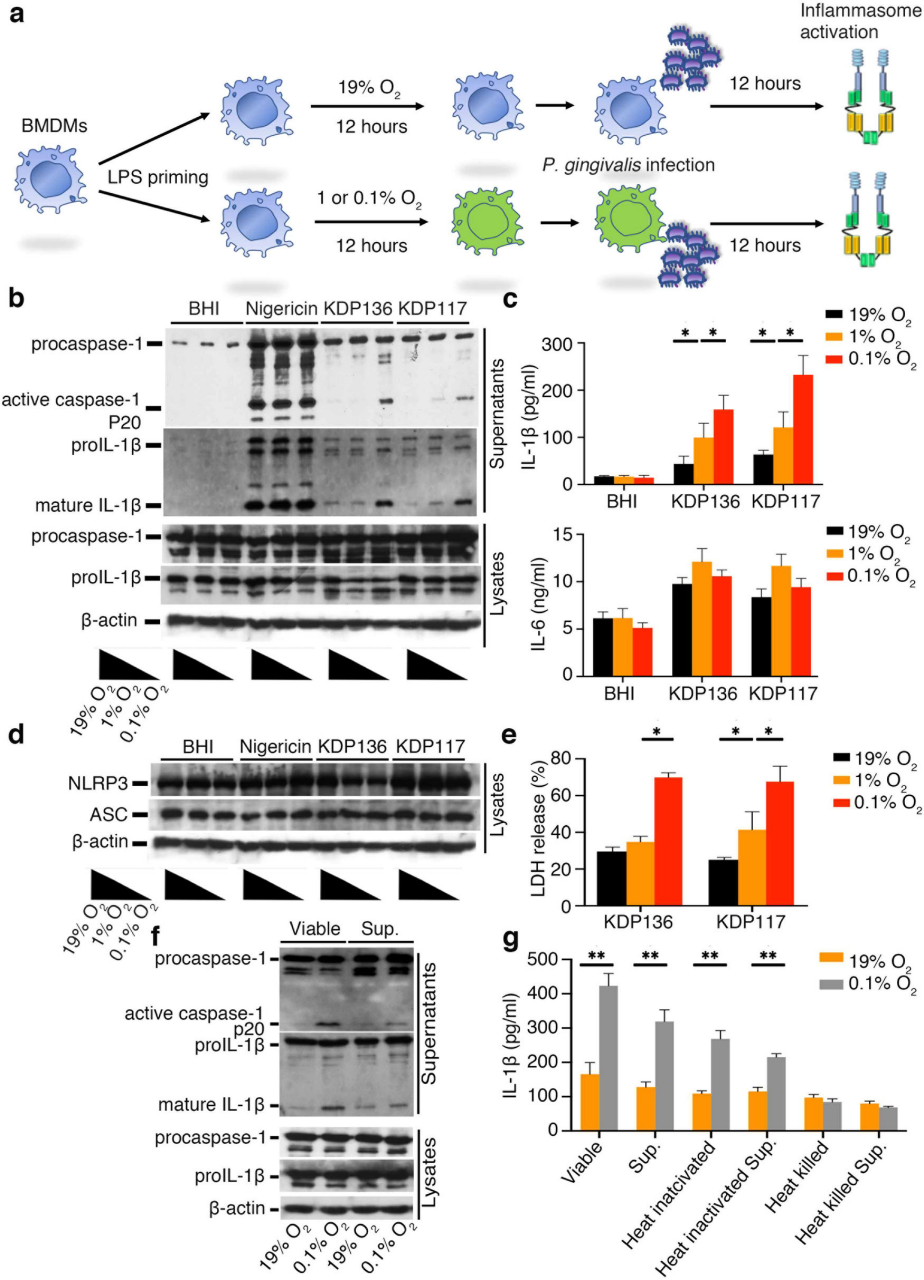
Inflammasome activation and cell death induced by secreted proteins from *Pg* are upregulated under hypoxia. **a** Schematic representation of *Pg* infection of BMDMs followed by incubation under normoxia (19% O_2_) or hypoxia (0.1% O_2_). **b**–**e** LPS-primed (200 ng/ml) BMDMs were incubated under normoxia or hypoxia for 12 h and then infected with *Pg* KDP136 or KDP117 under normoxia or hypoxia for an additional 12 h. As a control, 5 μM nigericin was added to LPS-primed BMDMs (200 ng/ml) to activate the NLRP3 inflammasome after incubation under normoxia (19% O_2_) or hypoxia (0.1% O_2_) for 30 min. Brain heart infusion (BHI) medium was used as a negative control for inflammasome activation. Cell lysates and culture supernatants were harvested 12 h post-infection (hpi). IL-1β and IL-6 release in the culture supernatants were measured by ELISA. The pro-form of caspase-1 (procaspase-1, 45 kDa), pro-form of IL-1β (pro-IL-1β, 31 kDa), NLRP3, ASC, and β-actin in the cells, along with the active form of caspase-1 P20 (active caspase-1, 20 kDa) and mature form of IL-1β (17 kDa) in the culture supernatants, were analyzed by immunoblot. Cell death was assessed by LDH release. IL-1β and IL-6 in the supernatants were also measured by ELISA. **f**, **g** *Pg* KDP117 (viable) culture or bacterial culture supernatants (Sup.), heat-inactivated bacterial culture (65°C), supernatants from heat-killed bacterial cultures (heat-killed sup.), or heat-killed bacterial culture (95 °C) or supernatants from heat-killed bacterial culture (heat-killed sup.) were added to LPS-primed (200 ng/ml) BMDMs. Cell lysates and culture supernatants were harvested at 12 hpi. The pro-form of caspase-1 (procaspase-1, 45 kDa), pro-form of IL-1β (pro-IL-1β, 31 kDa), and β-actin in the cells, along with the active form of caspase-1 P20 (active caspase-1, 20 kDa) and mature form of IL-1β (17 kDa) in the culture supernatants, were analyzed by immunoblot. IL-1β and IL-6 release in the culture supernatants were measured by ELISA. Blots are representative of three independent experiments. **b**, **d**, **f** Data are shown as mean ± SD of triplicates and represent the results of three independent experiments. **c**, **e**, **g** *P < 0.05 and **P < 0.01 indicate statistically significant differences, as determined using a T-test.

To investigate the effect of hypoxia on inflammasome activation, bone marrow-derived macrophages (BMDMs) were infected with *Pg* KDP136, lacking gingipains (RgpA, RgpB, and Kgp), or *Pg* KDP117, lacking PorT, under normoxic (19% O_2_) and hypoxic conditions. Some studies reported average oxygen concentration in periodontal pockets was around 1.5% and the lowest one was under 0.7% and we set 2 kinds of oxidative concentration (1 and 0.1%) in the first screening [34–36]. Figure 1a describes a schematic diagram of the in vitro study. Since gingipains secreted by *Pg* interfere with the detection of activated caspase-1 and mature IL-1β, we used gingipain-deficient strains KDP136 and KDP117. Under hypoxia (1% or 0.1% O_2_), these strains induced caspase-1 activation and IL-1β release, but not IL-6, compared to normoxia (Fig. 1b, c).

We also conducted immunohistochemical analysis to assess ASC speck formation, a hallmark of inflammasome activation, using the ATCC33277 strain of *Pg*, which secretes gingipains [13]. Under hypoxia, ASC speck formation increased following infection with ATCC33277, but not after treatment with nigericin, a positive control for NLRP3-mediated inflammasome activation (Fig. S1a, b). Protein levels of inflammasome components NLRP3 and ASC were similar between normoxic and hypoxic conditions (Fig. 1d).

Next, we assessed cytotoxicity by measuring lactate dehydrogenase (LDH) release, which was elevated under hypoxia, particularly at 0.1% O_2_ (Fig. 1e). To confirm that inflammasome activation was specifically induced by *Pg*, we tested known inflammasome activators, including nigericin, silica (NLRP3), *Salmonella enterica* serovar *Typhimurium* (NLRC4), poly (dA:dT) (AIM2), and *Escherichia coli* (caspase-11) [37, 38]. None of these treatments significantly increased IL-1β release under hypoxia compared to normoxia (Fig. S1c). We have shown that bacterial secreted or released components from *Pg* are previously involved in inflammasome activation [20]. To determine the bacterial components responsible for inflammasome activation under hypoxia, we exposed BMDMs to *Pg* culture supernatants, heat-inactivated bacteria (65 °C for 30 min), and heat-killed bacteria (100 °C for 30 min). Caspase-1 activation and IL-1β release increased with the bacterial culture supernatants and heat-inactivated bacteria but not with heat-killed bacteria under hypoxia (Fig. 1f, g). These findings suggest that components secreted or released by *Pg*, excluding lipopolysaccharide (LPS), enhance inflammasome activation and cytotoxicity under hypoxic conditions.

To investigate the role of NLRs in inflammasome activation induced by *Pg* under hypoxia, we assessed caspase-1 activation and IL-1β release in BMDMs from wild-type, NLRP3-deficient, and AIM2-deficient mice. In NLRP3-deficient BMDMs, neither caspase-1 activation nor IL-1β release was observed under normoxic or hypoxic conditions. In contrast, both responses were evident in BMDMs from AIM2- and caspase-11-deficient mice infected with *Pg* KDP117 (Fig. S2a–d). These results indicate that *Pg* infection triggers NLRP3-mediated inflammasome activation via the canonical pathway, regardless of oxygen levels.

### TLR4-TRIF-MyD88 activation is essential for enhancement of inflammasome activation induced by *Pg* under hypoxia

**Fig. 2:**
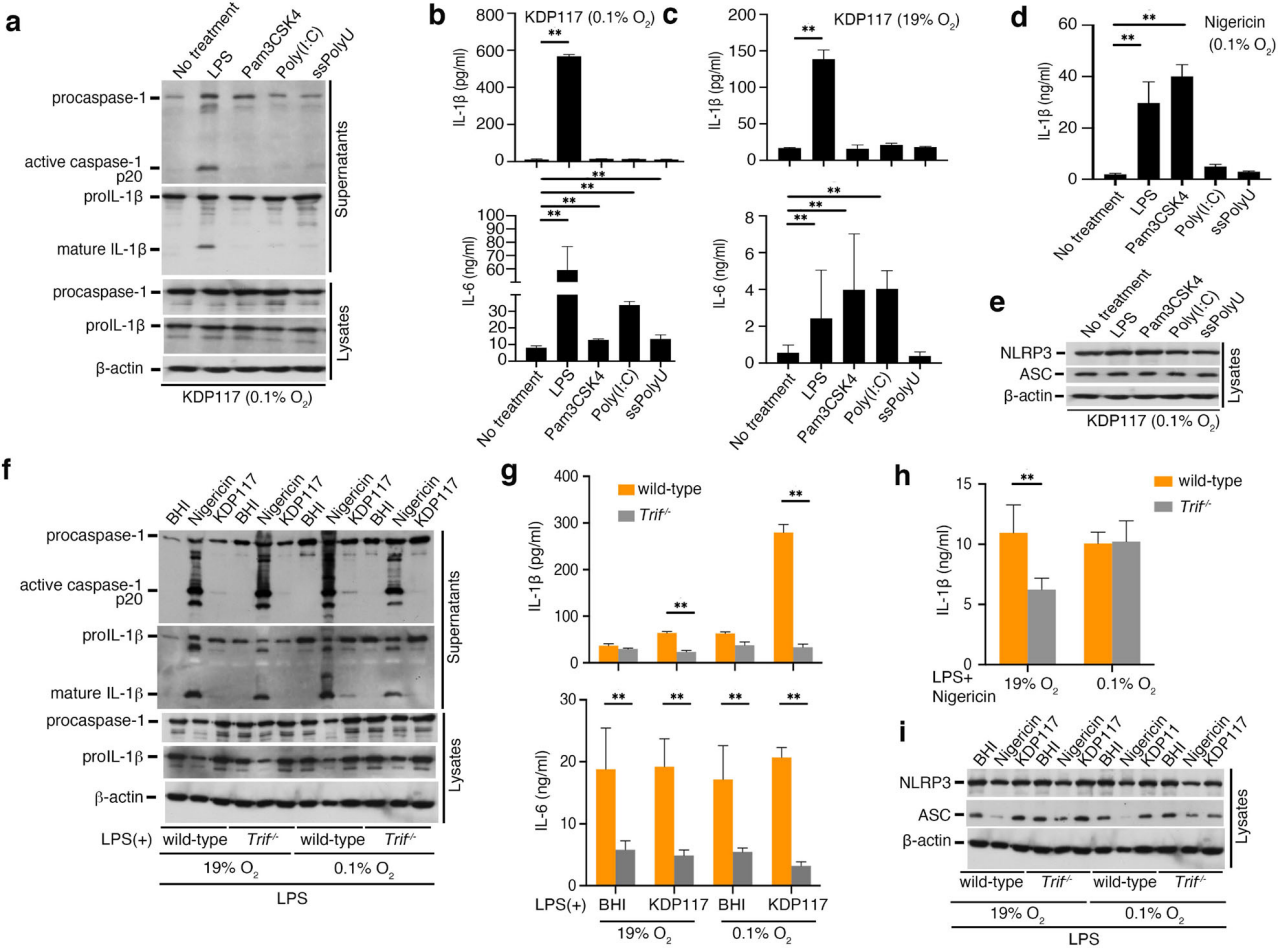
The TLR4-TRIF axis is essential for the enhanced inflammasome activation induced in macrophages by *Pg* infection under hypoxia. **a**, **b**, **c**, **e** LPS (200 ng/ml), Pam3CSK4 (1 μg/ml), Poly(I:C) (1 μg/ml), and ssPolyU (1 μg/ml)-primed BMDMs under normoxia (21%O_2_) or hypoxia (0.1% O_2_) for 12 h were infected with *Pg* KDP117. Cell lysates and culture supernatants were harvested at 12 hpi. Procaspase-1 (45 kDa), pro-IL-1β (31 kDa), and β-actin in the cells, and the active form of caspase-1 subunit P20 (20 kDa) and the mature form of IL-1β (17 kDa) in the culture supernatants were analyzed by immunoblot. IL-1β release in the culture supernatants was measured by ELISA. **d** 200 ng/ml LPS-, 1 μg/ml Pam3CSK4-, 1 μg/ml Poly(I:C)-, and 1 μg/ml ssPolyU- primed BMDMs under hypoxia for 12 h were treated with 5 μΜ nigericin. Supernatants were harvested 30 min posttreatment, and IL-1β release was measured by ELISA. **f**, **g**, **i** LPS-primed BMDMs (200 ng/ml) from wild-type or TRIF-deficient (*Trif*^*−/*^^−^) mice incubated under normoxia or hypoxia for 12 h were infected with *Pg* KDP117 under normoxia or hypoxia. Subsequently, 5 μΜ nigericin was added as a control to activate the NLRP3 inflammasome, and BHI medium was added as a negative control. Cell lysates and supernatants were harvested at 12 hpi. Procaspase-1 (45 kDa), pro-IL-1β (31 kDa), and β-actin in the cells along with subunits of the active form of caspase-1 P20 (20 kDa) and the mature form of IL-1β (17 kDa) in the culture supernatants were analyzed by immunoblot. IL-1β release in the culture supernatants was measured by ELISA. h LPS-primed BMDMs (200 ng/ml) from wild-type or TRIF-deficient (*Tri*^*f−/−*^*)* mice were treated with 5 μM nigericin under normoxia or hypoxia. Supernatants were harvested 30 min posttreatment, and IL-1β release in the culture supernatants was measured by ELISA. Blots are representative of three independent experiments. **a**, **e**, **f**, **i** Data are shown as means ± SD of triplicates and represent the results of three independent experiments. **b**, **c**, **d**, **g**, **h** **P < 0.01 indicates a statistically significant difference as determined using a T-test.

Previous study demonstrated that LPS priming enhances *Pg*-triggered IL-1β release [20]. To investigate the role of LPS priming in the enhancement of inflammasome activation induced by *Pg* under hypoxia, we assessed four TLR ligands (Pam3CSK4, LPS, poly(I:C), and ssPolyU) for their ability to prime BMDMs infected with *Pg* KDP117 under hypoxic conditions. Caspase-1 activation and IL-1β release were detected in LPS-primed BMDMs but not in those primed with other ligands, whereas IL-6 release was observed with all four ligands under hypoxia (Fig. 2a, b). We also observed LPS priming induces IL-1β secretion with *Pg* infection under nomoxia (Fig. 2c).

We also examined IL-1β release from BMDMs primed with TLR ligands and treated with nigericin under hypoxia. IL-1β release occurred in BMDMs primed with LPS, Pam3CSK4, and poly I:C (Fig. 2d). Additionally, NLRP3 and ASC protein levels in BMDMs infected with *Pg* KDP117 under hypoxia remained unchanged, regardless of TLR ligand priming (Fig. 2e).

These results indicate that the enhancement of inflammasome activation induced by *Pg* under hypoxia involves TLR4 signaling, which activates the TRIF and MyD88 pathways [39]. To confirm the roles of TRIF and MyD88 in this process, we used BMDMs derived from TRIF- and MyD88-deficient mice. Under hypoxia, caspase-1 activation was not enhanced in BMDMs from either TRIF- or MyD88-deficient mice (Figs. 2f and S3a). IL-1β and IL-6 release were similarly unaffected in TRIF-deficient BMDMs and undetectable in MyD88-deficient BMDMs (Figs. 2g and S3b).

Furthermore, IL-1β release in BMDMs from TRIF-deficient mice was not enhanced under hypoxia, and no IL-1β was detected in BMDMs from MyD88-deficient mice (Figs. 2h and S3c). The expression levels of ASC and NLRP3 under hypoxia and normoxia were similar in TRIF-deficient BMDMs. However, NLRP3 and Pro-IL-1β levels were reduced in MyD88-deficient BMDMs, as the MyD88-NFκB axis regulates the expression of both proteins (Figs. 2i and S3d).

Therefore, these results demonstrate that LPS-mediated TRIF and MyD88 activation through TLR4 is critical for the enhancement of inflammasome activation induced by *Pg* infection under hypoxia

### TRIF regulates HIF-1α expression levels

**Fig. 3:**
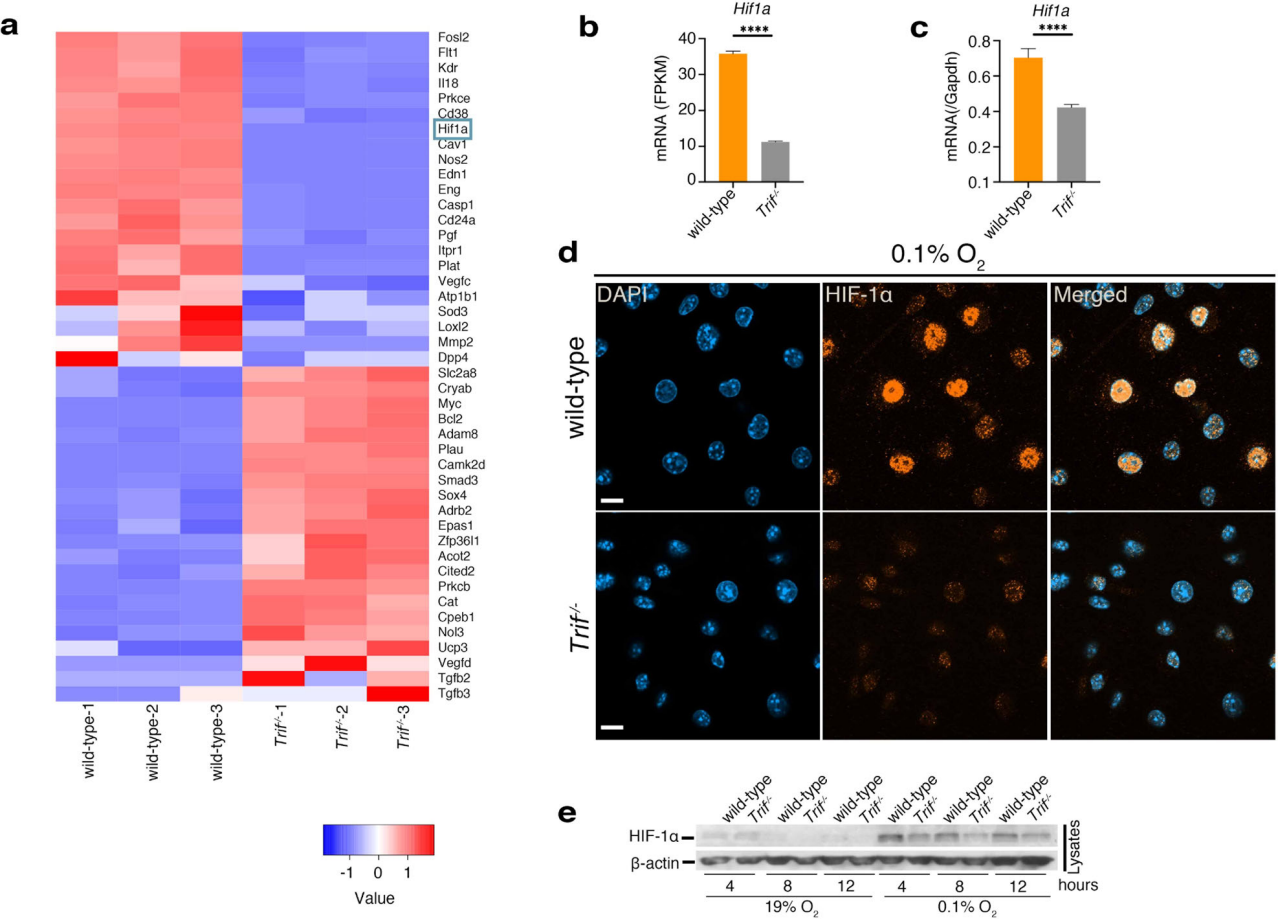
HIF-1α is upregulated by the TRIF signaling pathway with TLR4 ligand priming under hypoxia. **a**–**d** LPS-primed BMDMs (200 ng/ml) from wild-type or *Trif*^*−/−*^ mice were incubated under hypoxia (0.1% O_2_). Cells were harvested at 8 h postincubation for RNA extraction or fixation. A heat map shows hypoxia response genes significantly altered (*p* < 0.05) by 2-fold relative to *Trif*^*−/−*^ BMDMs. *N* = 3 samples per condition in RNA-seq. FPKM for HIF-1α mRNA was obtained from RNA-seq, and HIF-1α mRNA levels were measured by real-time PCR. HIF-1α was stained using a Cy3-labeled anti-HIF-1α antibody (orange) and 4′,6-diamidino-2-phenylindole (DAPI, blue) for nuclear visualization. Merged images are shown. Scale bar, 20 μm. e, LPS-primed BMDMs (200 ng/ml) from wild-type or *Trif*^*−/−*^ mice were incubated under hypoxia. The cells were harvested at 2, 4, and 8 h post-incubation. HIF-1α and β-actin in the cells were analyzed by immunoblot. Blots and stained images are representative of three independent experiments. **d**, **e** Data are presented as means ± SD of triplicates. **b**, **c** ***P < 0.0001 indicates statistically significant differences, as determined using a T-test.

To analyze TRIF activity under hypoxia, we examined the expression levels of genes related to the hypoxic response in BMDMs from wild-type and TRIF-deficient mice through RNA sequencing (Fig. 3a and Table S1). We focused on HIF-1α expression, assessing mRNA levels using both Fragments Per Kilobase of exon per Million mapped reads (FPKM) from RNA sequencing and real-time PCR. Both methods revealed a significant decrease in HIF-1α mRNA expression under hypoxia in BMDMs from TRIF-deficient mice (Fig. 3b, c). This decrease was also observed under normoxia (Fig. S4a).

Additionally, we assessed HIF-1α nuclear translocation under hypoxia in BMDMs from both wild-type and TRIF-deficient mice using immunohistochemistry. Translocation was diminished in BMDMs from TRIF-deficient mice (Fig. 3d). Under normoxia, HIF-1α translocation was absent in both wild-type and TRIF-deficient BMDMs (Fig. S4b). Furthermore, immunoblotting analysis confirmed stable HIF-1α levels in BMDMs from TRIF-deficient mice after 4, 8, and 12 h of hypoxia compared to wild-type mice (Fig. 3e). However, *Pg* KDP117 infection did not alter stable HIF-1α levels in BMDMs (Fig. S4c). These findings suggest that TRIF regulates HIF-1α expression under both normoxia and hypoxia, affecting its nuclear translocation without *Pg* infection.

### HIF-1α is essential for enhancement of inflammasome activation induced by *Pg* infection under hypoxia

**Fig. 4:**
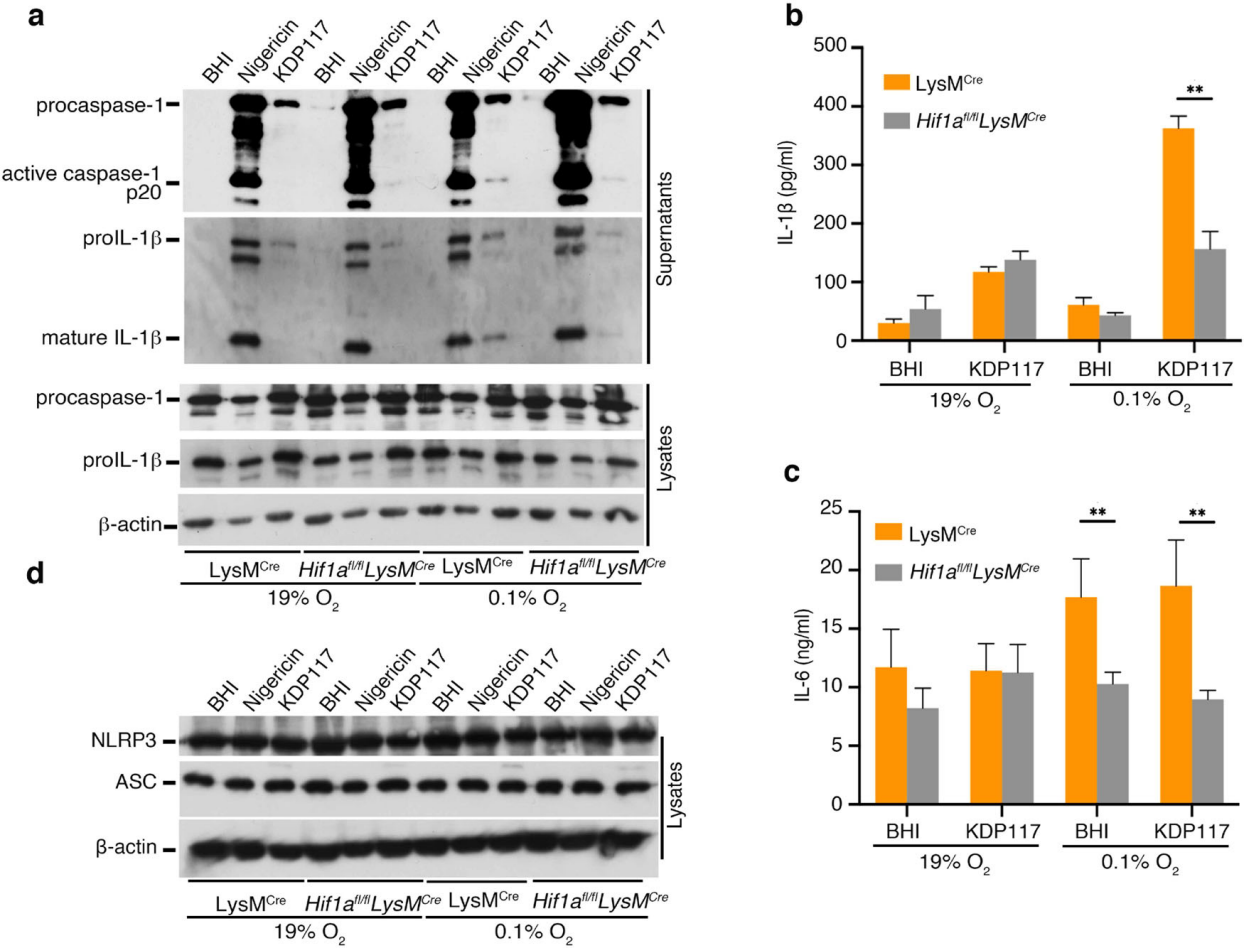
HIF-1α controls the enhanced inflammasome activation induced by *Pg* infection under hypoxia. LPS-primed BMDMs (200 ng/ml) from LysM-Cre transgenic line (LysM^Cre^) or myeloid cell-specific HIF-1α deficient (*Hif1a*^*fl/fl*^*LysM*^*Cre*^) mice were incubated under normoxia (19% O_2_) or hypoxia (0.1% O_2_) for 12 h and infected with *Pg* KDP117. Cell lysates and supernatants were harvested at 12 hpi and analyzed for procaspase-1, pro-IL-1β, β-actin, active-caspase-1, mature IL-1β, NLRP3, and ASC by immunoblot. IL-1β and IL-6 levels in the supernatants were measured by ELISA. Blots are representative of three independent experiments. **a**–**d** Data are shown as means ± SD of triplicates. **b**, **c** **P < 0.01 indicates statistically significant differences, as determined using a T-test.

To evaluate the role of HIF-1α in inflammasome activation under hypoxia during *Pg* infection, we used myeloid cell-specific HIF-1α-deficient mice by crossing *HIF-1α*^*flox/flox*^ mice with the *LysM-Cre* transgenic line (*Hif1a*^*fl/fl*^*LysM*^*Cre*^ mice) [40]. Caspase-1 activation and IL-1β release induced by *Pg* KDP117 infection under hypoxia were enhanced in BMDMs from *LysM*^*Cre*^ mice but not in those from *Hif1a*^*fl/fl*^*LysM*^*Cre*^ mice (Fig. 4a, b).

We also measured IL-6 secretion in these BMDMs and observed that IL-6 release was reduced under hypoxia in *Hif1a*^*fl/fl*^*LysM*^*Cre*^-derived BMDMs, regardless of infection with *Pg* KDP117 (Fig. 4c). However, immunoblot analysis revealed no changes in NLRP3 and ASC protein levels (Fig. 4d). To further assess the involvement of HIF-1α, we used KC7F2, a HIF-1α inhibitor, and observed that caspase-1 activation and IL-1β release under hypoxia were suppressed in BMDMs treated with KC7F2 (Fig. S5a, b). These findings suggest that HIF-1α plays a crucial role in accelerating inflammasome activation in BMDMs during *Pg* infection under hypoxia.

### Myeloid cell-specific HIF-1α drives the progression of EAE induced by *Pg* infection

Previous studies have shown that inflammasome activation or *Pg* infection exacerbates EAE in a mouse model of autoimmune disease characterized by impaired proliferation of memory T-cells in the lymph nodes [29]. We induced EAE in C57BL/6 mice through oral infection with *Pg* ATCC33277 and *Prevotella intermedia* (*P. intermedia*) ATCC25611, one of the periodontitis-associated bacteria. Mice administered carboxymethyl cellulose (CMC) orally were used as a negative control (Fig. S6a). The clinical scores of the *Pg*-infected mice were significantly higher than those of the *P. intermedia*-infected or CMC-treated mice (Fig. S6b).

**Fig. 5:**
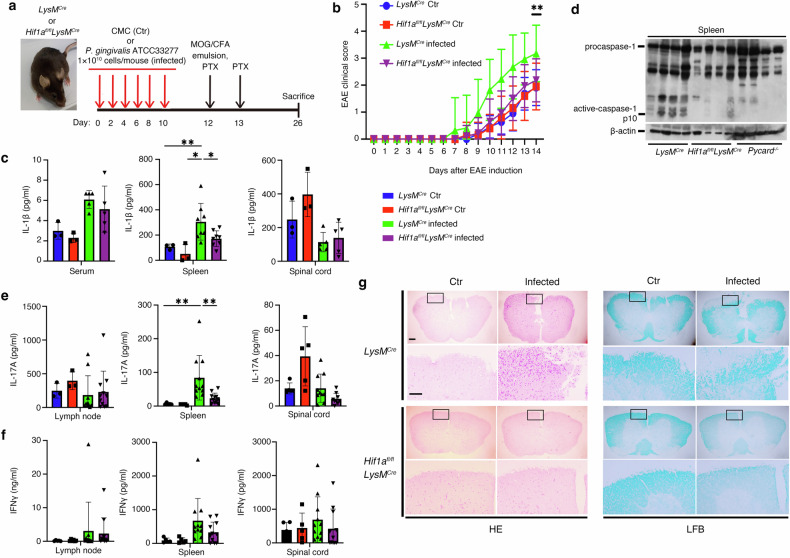
Oral *Pg* infection exacerbates neurological symptoms and inflammasome activation in a HIF-1α-dependent manner. *LysM*^*Cre*^ or *Hif1a*^*fl/fl*^*LysM*^*Cre*^ mice were orally infected with *Pg* ATCC33277 or administered carboxymethyl cellulose (CMC) orally on days 0, 2, 4, 6, 8, and 10. EAE development was induced by MOG35-55 and pertussis toxin in mice. Clinical scores were monitored for 14 days. Serum, spleen, spinal cord, and lymph nodes were collected on day 14 for immunoblot and/or cytokine analysis. *LysM*^*Cre*^ + CMC (*LysM*^*Cre*^ Ctr), *Hif1a*^*fl/fl*^*LysM*^*Cre*^ + CMC (*Hif1a*^*fl/fl*^*LysM*^*Cre*^ Ctr), *n* = 5 mice per group; *LysM*^*Cre*^ + *Pg* ATCC33277 (*LysM*^*Cr*^ infected), *n* = 17 mice; and *Hif1a*^*fl/fl*^*LysM*^*Cre*^ + *Pg* ATCC33277 (*Hif1a*^*fl/fl*^*LysM*^*Cre*^ infected), *n* = 14 mice. **a** Schematic of the EAE model. **b** Clinical score. **c** IL-1β release in the serum, spleen, and spinal cord was measured by ELISA. **d** Procaspase-1, the active subunit of caspase-1, and β-actin in the spleen were detected by immunoblot. **e** IL-17A release in the lymph node, spleen, and spinal cord was measured by ELISA. **f** IFNγ release in the lymph node, spleen, and spinal cord was measured by ELISA. **g** Pathological analysis of the spinal cord shows the representative area of inflammatory cellular infiltrate (hematoxylin and eosin, HE) and demyelination (Luxol fast blue, LFB). Areas enclosed within black squares are magnified on the right. Scale bar, 200 μm. **d** Data are presented as means ± SD of triplicates. *P < 0.05 and **P < 0.01 indicate statistically significant differences, determined by one-way ANOVA with Tukey’s test (**b**, **c**, **e**, **f**).

To identify the role of enhanced HIF-1α-mediated inflammasome activation induced by *Pg* infection in EAE progression, we elicited EAE in *LysM*^*Cre*^ and *Hif1a*^*fl/fl*^*LysM*^*Cre*^ mice, with or without *Pg* infection (Fig. 5a). The clinical scores of the *Hif1a*^*fl/fl*^*LysM*^*Cre*^ mice were lower than those of the *LysM*^*Cre*^ mice with *Pg* infection (Fig. 5b). Additionally, we detected higher levels of IL-1β in the serum, spleen, and spinal cord of *LysM*^*Cre*^ mice with *Pg* infection, while *Hif1a*^*fl/fl*^*LysM*^*Cre*^ mice had higher IL-1β levels only in the spleen (Fig. 5c). Notably, higher levels of IL-1β were also observed in the spleens of C57BL/6 mice infected with *Pg*, compared to those infected with *P. intermedia* or treated with CMC (Fig. S6c).

Strong caspase-1 activation was observed in the spleens of *LysM*^*Cre*^ mice, while only weak activation occurred in *Hif1a*^*fl/fl*^*LysM*^*Cre*^ mice with *Pg* infection. No activation was detected in Pycard-deficient (*Pycard*^*−/−*^) mice, which lack the ability to form the inflammasome complex (Fig. 5d). We then examined IL-17 and IFNγ levels in cells stimulated with ionomycin and phorbol 12-myristate 13-acetate (PMA) from the lymph nodes, spleen, and spinal cord. Although IL-17- or IFNγ-expressing CD4^+^ T-cell counts were not significantly different among the groups (data not shown), higher levels of IL-17 were found in the spleens of *LysM*^*Cre*^ mice with *Pg* infection, but not in their lymph nodes or spinal cords, compared to *Hif1a*^*fl/fl*^*LysM*^*Cre*^ mice or mice without infection (Fig. 5e). No significant differences in IFNγ levels were observed across the groups (Fig. 5f). Additionally, we also displayed decreased inflammatory cell infiltration in the spinal cord from *Hif1a*^*fl/fl*^*LysM*^*Cre*^ mice with *Pg* infection as well as demyelination compared to other groups (Fig. 5g). These data suggest that oral *Pg* infection triggers IL-1β and IL-17-mediated enhancement of HIF-1α-driven inflammasome activation in spleen myeloid cells, thereby aggravating EAE.

## Discussion

Our study revealed that the enhancement of NLRP3 inflammasome activation in macrophages induced by *Pg* infection under hypoxia aggravated EAE in a HIF-1α-dependent manner. We also found that TRIF regulates the mRNA expression and nuclear translocation of HIF-1α.

Nevertheless, periodontal pockets in periodontitis patients are hypoxic, and almost all previous reports are based on in vitro experiments conducted under normoxia rather than hypoxia. This study discovered that hypoxia significantly enhances NLRP3 inflammasome activation in macrophages infected with *Pg* or treated with secreted proteins from *Pg*. Moreover, inflammasome component protein levels, such as NLRP3, ASC, and caspase-1, do not change in BMDMs under hypoxia compared to normoxia.

We identified that the TLR4-TRIF-MyD88 axis is necessary to enhance inflammasome activation induced by *Pg* infection under hypoxia. One report mentioned that TRIF is essential for triggering caspase-11-mediated noncanonical NLRP3 inflammasome activation [41]. However, in this study, caspase-11 was not associated with inflammasome activation induced by *Pg* infection under hypoxia.

In this study, HIF-1α expression levels and nuclear translocation efficiency under hypoxia were downregulated in BMDMs derived from TRIF-deficient mice compared to wild-type mice. Additionally, HIF-1α deficiency in BMDMs suppressed the enhancement of inflammasome activation induced by *Pg* infection under hypoxia. Many previous studies have demonstrated the importance of HIF-1α in inflammation and cell death, including inflammasome activation [42, 43]. However, low HIF-1α expression was not associated with suppressed expression of inflammasome component proteins, such as ASC or NLRP3, under hypoxia. Additionally, NLRP3 and ASC protein levels are not altered in BMDMs from TRIF-deficient compared to wild-type mice. We consider that LPS priming before Pg infection induces enough expression level of these genes under normoxia and hypoxia with or without TRIF or HIF-1α deficiency. We observed a difference in the level of inflammasome activation between TRIF- and HIF-1α-deficient macrophages induced by *Pg* infection under hypoxia, suggesting that other factors may be involved in enhancing inflammasome activation via the TRIF pathway.

The EAE model is the most commonly used experimental model for human multiple sclerosis [29]. We found that oral *Pg* infection aggravated the progression of EAE in a HIF-1α-dependent manner, specifically in myeloid cells. Notably, IL-1β production and caspase-1 activation were increased in the spleen of infected mice compared to those in the serum and lymph nodes. Therefore, oral *Pg* infection directly or indirectly activates the inflammasome in spleen myeloid cells in a HIF-1α-dependent manner. However, since we could not detect *Pg* in the spleen after oral infection, the bacterial proteins may be transferred to the spleen via blood vessels, or inflammation from oral tissues may spread systemically, triggering inflammasome activation.

The association between EAE progression and HIF-1α remains controversial. Some studies suggest that HIF-1α in myeloid cells is not required for the development of EAE [44], while another report demonstrated that a HIF-1α transcription inhibitor suppresses EAE severity [45]. However, our results indicated an indirect relationship between HIF-1α and EAE progression in the context of *Pg* infection. This study provides new insights not only into inflammasome activation induced by *Pg* infection under hypoxia but also into the role of HIF-1α in both innate and adaptive immunity in the host.

## Materials and methods

### Ethics statement

All animal studies were approved by the Institutional Animal Care and Use Committee of the Institute of Science Tokyo (approval number: A2021-118A, A2023-160A). The experimental protocols covering the use of Living Modified Organisms, including bacterial mutants and gene-knockout mice, were approved by the Genetically Modified Organisms Safety Committee of the Institute of Science Tokyo (approval number: G2018-021C7). In the handling of *Pg* and *P. intermedia* strains under biosafety level, the Safety Control Committee approved two conditions for Pathogenic Microbes of the Institute of Science Tokyo (approval number: M22019-004C).

### Bacterial strains

*Pg* (ATCC33277) and *P. intermedia* (ATCC25611) were seeded on CDC anaerobe blood agar at 37°C for 5 days under anaerobic conditions (5% H_2_, 10% CO_2_, and 85% N_2_). The mutants of *P.* *gingivalis*, KDP117 lacking PorT (ΔporT), and KDP136 lacking all gingipains (RgpA, RgpB, and Kgp) were used. The mutant strains were maintained in enriched BHI broth supplemented with hemin, vitamin K. Bacterial culture supernatants were collected after 2 days of cultivation by centrifugation and filtration with 0.22 μm. *Salmonella enterica serovar Typhimurium* (SL1344) and *E. coli* (MC1061) were grown in Luria-Bertani broth under aerobic conditions at 37 °C for 1 day.

### Mice and preparation of macrophages

Wild-type C57BL/6 mice were purchased from Japan SLC (Tokyo, Japan). C57BL/6 background NLRP3-deficient, ASC-deficient, caspase-11-deficient, LysM-Cre transgenic, and myeloid cell-specific HIF-1α-deficient mice were housed in a pathogen-free facility. AIM2-deficient mice were provided by Dr. Kawaguchi (University of Tokyo). TRIF-deficient and MyD88-deficient mice were provided by Dr. Akira (Osaka University). Caspase-11-deficient mice were provided by Masahiro Yamamoto (Osaka University). Myeloid cell-specific HIF-1α-deficient mice were provided by Dr. Takeharu Sakamoto (Kansai Medical University). BMDMs were prepared from femurs and tibias of the above-mentioned mice and were cultured for 5–6 days in 10% FCS-RPMI 1640 supplemented with 30% mouse L-cell supernatant.

### Bacterial infection for immunoblotting

BMDMs seeded at a density of 1.3 × 10^6^ cells per well in 6-well plates were incubated for 12 h under 21, 1, or 0.1% O_2_ and infected with *Pg* at an MOI of 100 per cell or administered an equivalent volume of the corresponding bacterial culture supernatant under 21, 1, or 0.1% O_2_ for 12 h and the cells were lysed with lysis buffer (1% NP40). The culture supernatants were precipitated by adding 8% trichloroacetic acid. The samples were loaded onto 15% or 10% SDS-PAGE, and proteins were detected using anti-caspase-1 (Adipogen #AG-20B-0042-C100), IL-1β (R&D #AF-401-NA), NLRP3 (Adipogen #AG-20B-0006-C100), ASC Adipogen #AG-37B-0001-C100), HIF-1α (Cell signaling #3716), and β-actin (Merck #MAB1501) antibodies, respectively.

### Bacterial infection for ELISA and LDH release assay

BMDMs seeded at a density of 3.5 × 10^5^ cells per well in 24-well plates were incubated under 21, 1, or 0.1% O_2_ and infected with *Pg* at an MOI of 100 per cell or administered an equivalent volume of the corresponding bacterial culture supernatant, heat-inactivated bacteria, heat-killed bacteria, or culture supernatant from heat-killed bacteria under 21, 1, or 0.1% O_2_. At 12 h incubation after infection, the culture supernatants were collected, and the released cytokines were quantified using ELISA. The lactate dehydrogenase (LDH) activity in the culture supernatants was measured using a CytoTox 96 assay kit (Promega) in accordance with the manufacturer’s protocol.

### Immunofluorescence

BMDMs seeded at a density of 6.5 × 10^5^ cells per well in 6-well plates were incubated under 21 or 0.1% O_2,_ and infected with or without *Pg* at an MOI of 100 per cell at the indicated post-infection times. The infected BMDMs were fixed, immunostained as described previously [46], and then analyzed using a laser-scanning microscope (LSM-800; Carl Zeiss).

### Induction of EAE with bacterial infection

C57BL/6 wild-type, *LysM-Cre* transgenic, or *HIf1a*^*fl/fl*^*LysM*^*Cre*^ female mice between 10 and 13 weeks old were orally infected with *Pg* ATCC33277 (1 × 10^10^ cells/mouse), *P. intermedia* ATCC25611 (1 × 10^10^ cells/mouse) suspended in 50 μl of 2% carboxymethylcellulose (CMC) or 50 μl of CMC at 2-day intervals over a 12-day period. The mice were given 200 μg of myelin oligodendrocyte glycoprotein (MOG) 35-55 emulsified in Complete Freund’s Adjuvant (CFA) by subcutaneous injection and Pertussis toxin (PTX) by intraperitoneal on day 12. PTX toxin was also injected day 13, and this protocol is from previous study [47]. EAE was scored on days 0 to 14 or 15 after EAE induction as follows website: https://hookelabs.com/services/cro/eae/MouseEAEscoring.html.

### IL-1β detection for EAE model

Spleens, spinal cords, or lymph nodes were collected and incubated in liquid nitrogen. Cold tissues were destroyed using SK mill (TOKKEN, Inc.), and were added to homogenized buffer (0.1% NP-40). The supernatants were collected and centrifuged at 13,000 rpm at 4 °C. The supernatants were used as samples in ELISA analyses of IL-1β.

### IL-17 and IFNγ detection for EAE model

Spleens, spinal cords, or lymph nodes were collected in RPMI containing HEPES. Cells from tissues were stimulated by 50 ng/ml phorbol 12-myristate 13-acetate (PMA) and 500 ng/ml ionomycin for 24 h. The supernatants were used as samples in ELISA analyses of IL-17 or IFNγ.

### Histology

The spinal cords were collected from EAE mice on day 14. The samples were fixed in 4% paraformaldehyde overnight, paraffin-embedded, and sectioned (3 μm). Hematoxylin and eosin Y (H&E) staining and Luxol Fast Blue were performed using the standard protocol to visualize the infiltration of inflammatory cells into axonal myelin.

### Statistical analysis

All the data are presented as the mean ± SD of at least three determinations per experimental condition. All the experiments were performed at least three times, and representative results are shown in the figures. Statistical analyses were performed using unpaired two-tailed Student *t*-tests or a one-way ANOVA followed by the Tukey test. Differences were considered significant at a *p* value of <0.05, 0.01, or 0.001.

## Supplementary information

Supplementary Figs table legends.pdf

Uncropped images

Table S1

## Data Availability

The data generated during the current study are available from the corresponding author upon reasonable request.
