# Supplementary material for: Hypoxia drives progression of multiple sclerosis by enhancing the inflammasome activation in macrophages with Porphyromonas gingivalis infection
Source: Cell Death Discov. 2025 Jun 10;11:271. doi: 10.1038/s41420-025-02548-z (PMC12152135; doi:10.1038/s41420-025-02548-z)
Supplement: Supplementary file 1 — Supplementary Figs table legends.pdf [file 41420_2025_2548_MOESM1_ESM.pdf]

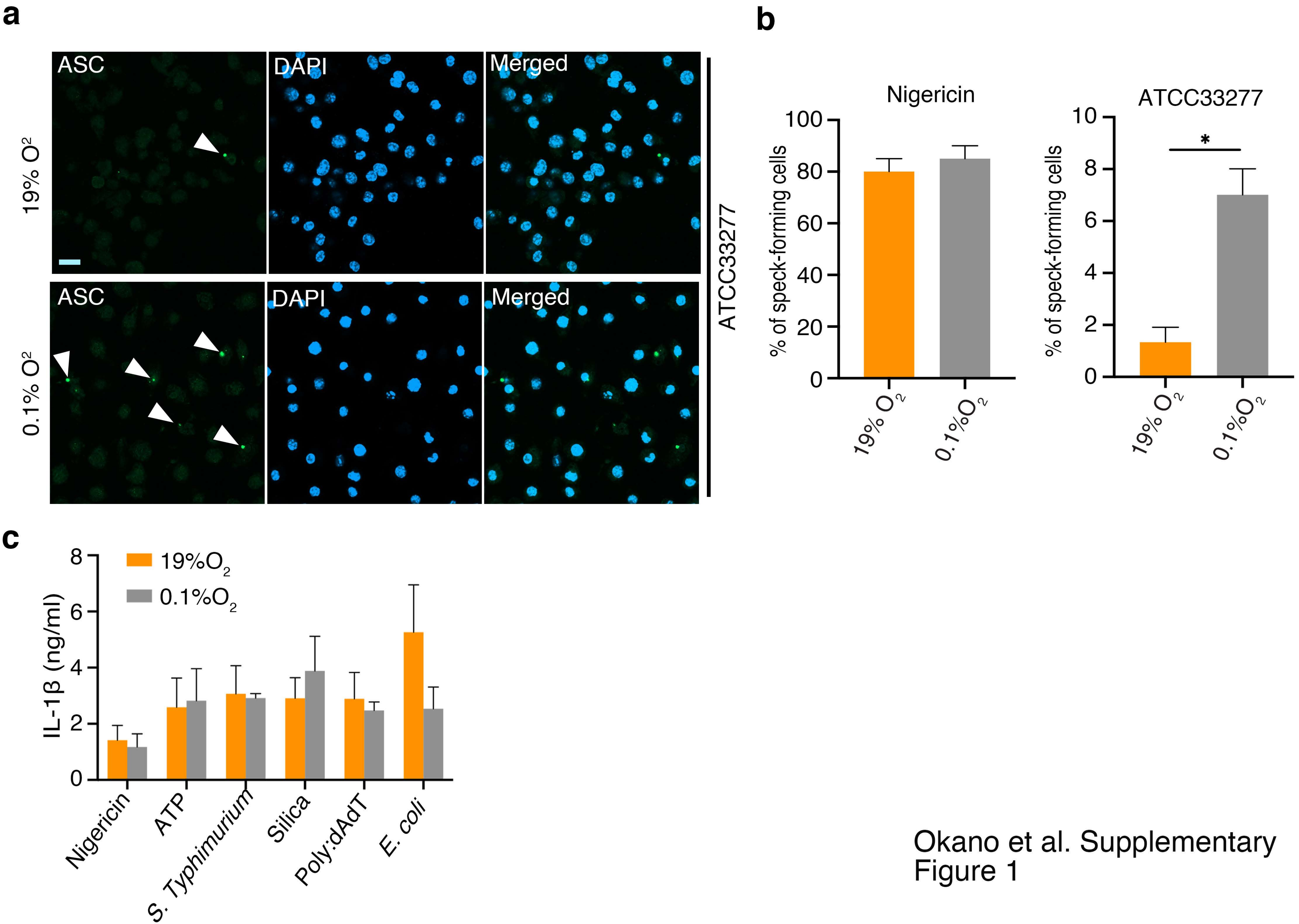

Okano et al. Supplementary Figure 1

Okano et al. Supplementary Figure 2

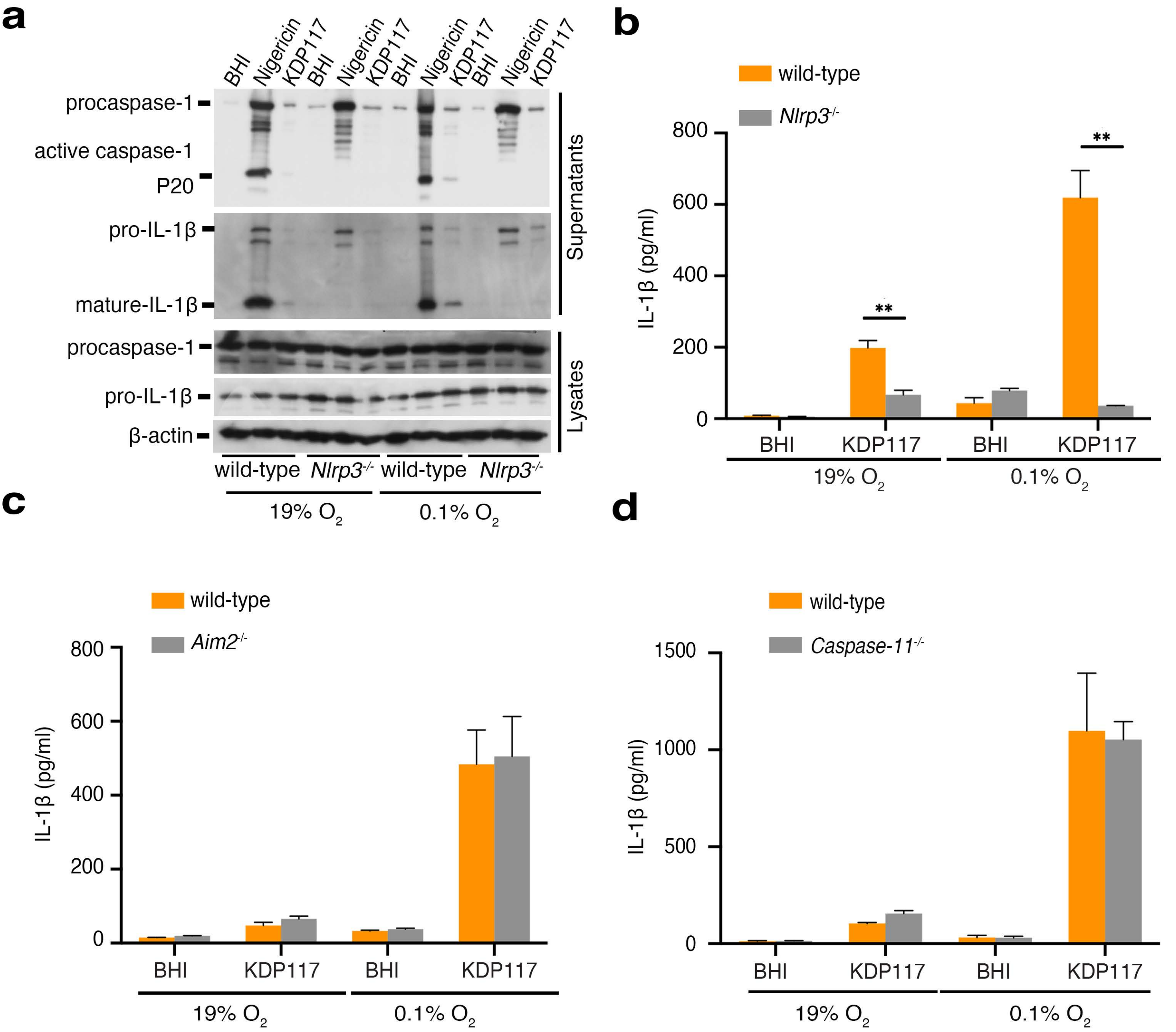

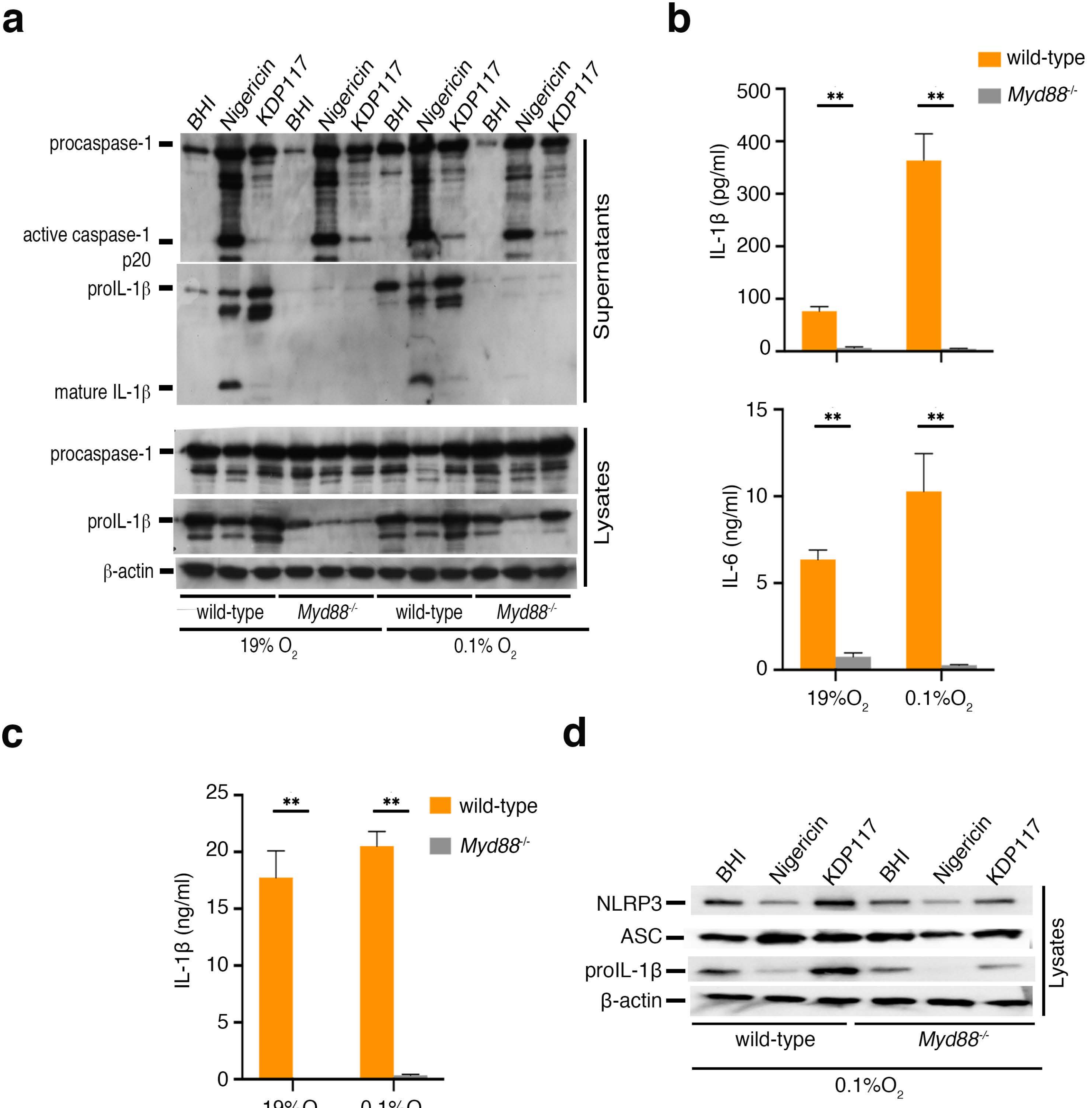

Okano et al. Suppramentary Data Fig. 3

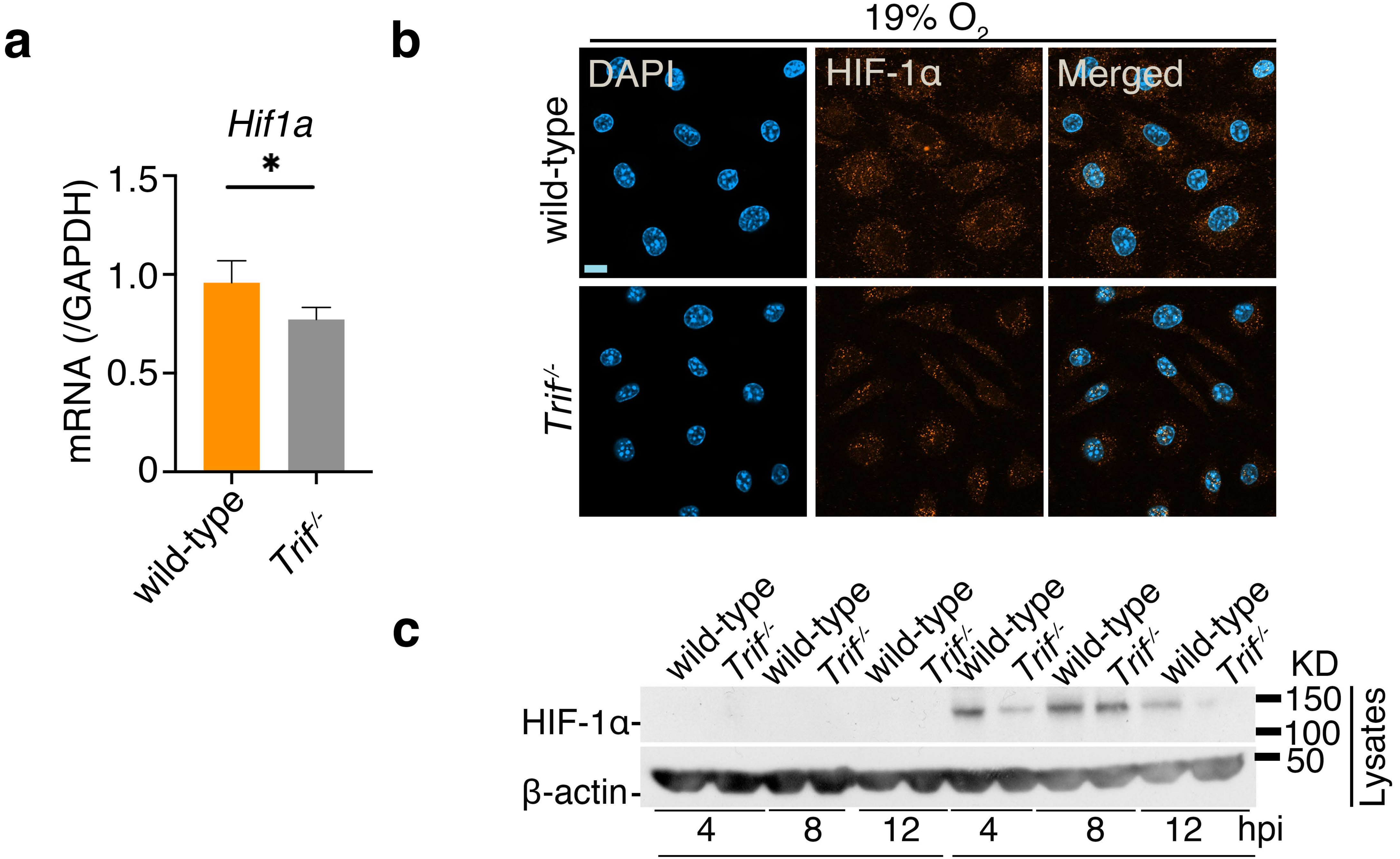

Okano et al. Supplementary Fig 4.

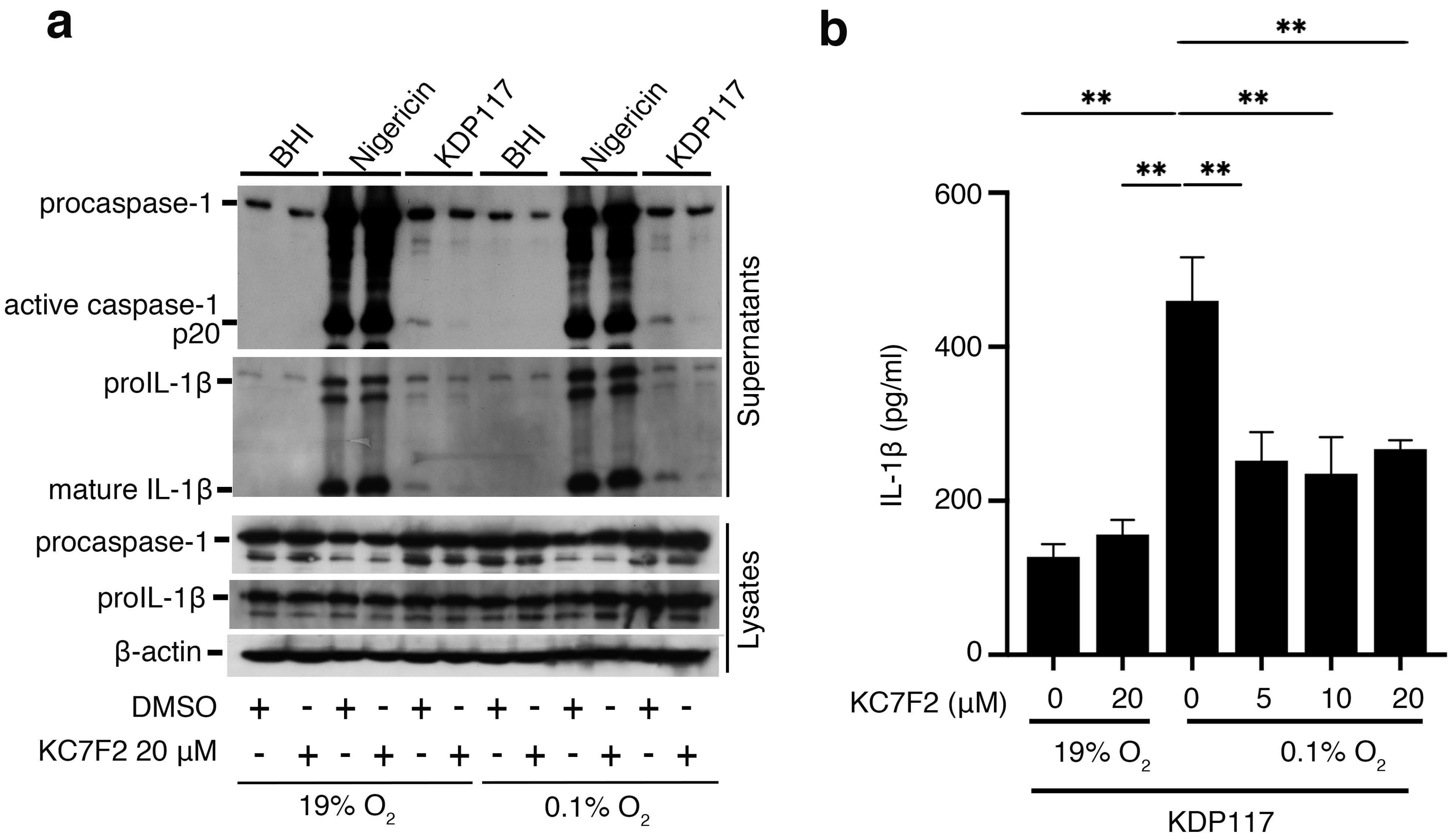

Okano et al. Supplementary
Figure 5

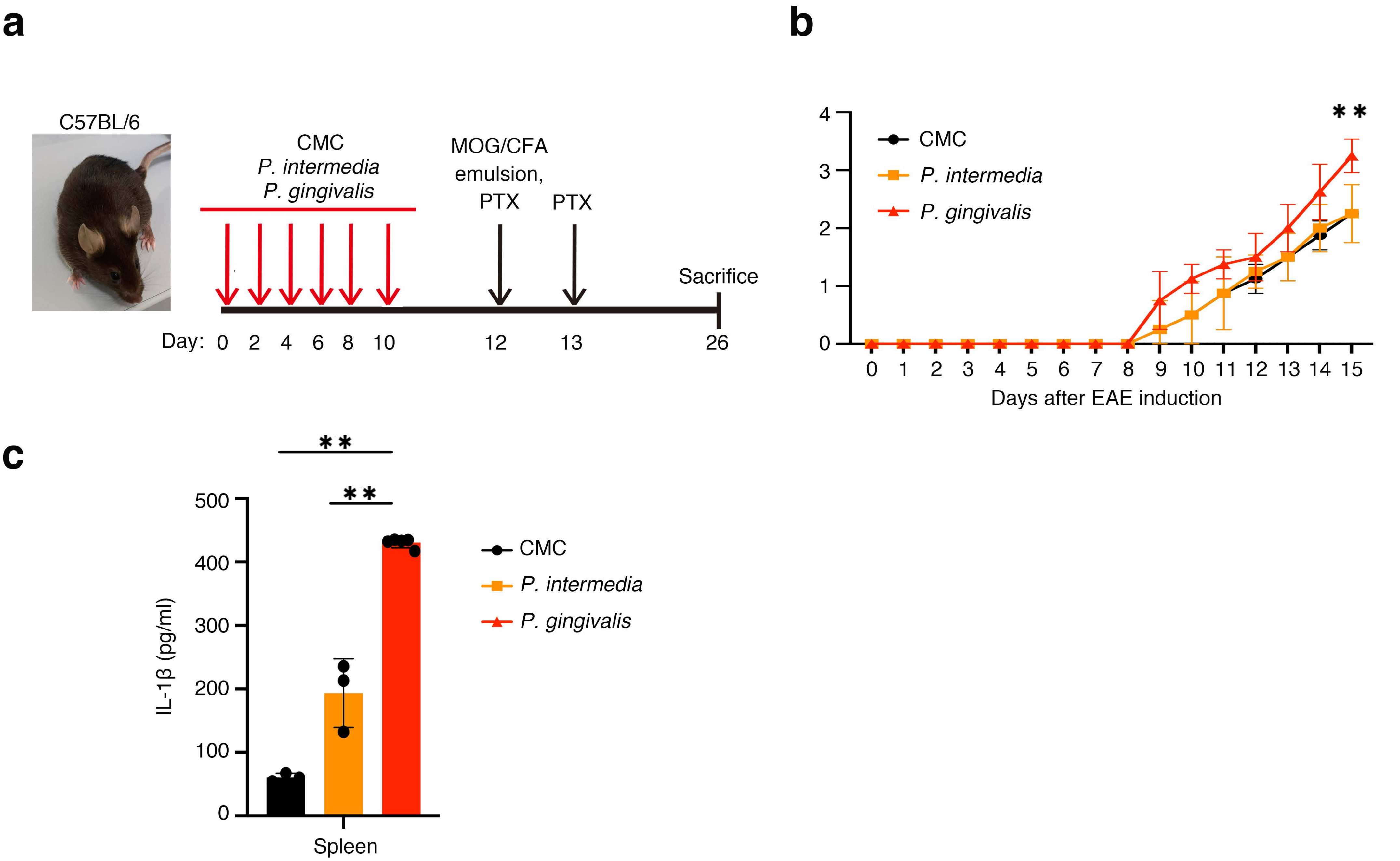

Okano et al Supplementary Figure 6

Supplementary figure legends

Fig. S1: Inflammasome activation and inflammatory cytokines release induced by *P. gingivalis* infection under hypoxia are enhanced in a gingipains-independent manner.

a, 200 ng/ml LPS-primed BMDMs were incubated under normoxia (19% O_2) or hypoxia (0.1% O_2) for 12 hours and infected with *P. gingivalis* ATCC33277 under normoxia or hypoxia for 12 hours.

b, 5 μM nigericin was added to the BMDMs as a control to activate the NLRP3 inflammasome after incubation under normoxia or hypoxia for 30 minutes. ASC speck formation (arrowheads) was analyzed by immunostaining. Scale bar, 20 μm. ASC was stained using Alexa fluor 488 (green), while the nuclei were stained using DAPI (blue).

c, 200 ng/ml LPS-primed BMDMs were incubated under normoxia (19% O_2) or

hypoxia (0.1% O_2) for 12 hours and treated with 5 μM nigericin for 30 minutes, 25 mM ATP for 1 hour, 70 μg/ml silica 12 hours, transfected Poly:dAdT for 6 hours, and infected *Escherichia coli* (*E. coli*) for 12 hours. IL-1β release in the culture supernatants was measured by ELISA.

Data shown are the means ± SD of triplicates and represent the results of three independent experiments. ∗P <0.01 indicates a statistically significant difference as determined using a T-test. (b,c)

Fig. S2: Enhancement of inflammasome activation induced by *P. gingivalis* infection under hypoxia is not dependent on AIM2, or caspase-11.

200 ng/ml LPS-primed BMDMs derived from wild-type, NLRP3-deficient (*Nlrp3*$^{-/-}$), AIM2-deficient (*AIM2*$^{-/-}$), or Caspase-11-deficient (*Caspase-11*$^{-/-}$) mice were incubated

under normoxia (19% O_2), or hypoxia (0.1% O_2) for 12 hours and infected with *P. gingivalis* KDP117 under normoxia or hypoxia for 12 hours. a, The procaspase-1, pro-IL-1β, and β-actin in the cells, subunits of the active-form of active-caspase-1 and mature IL-1β in the culture supernatants were analyzed by immunoblot. b-d, IL-1β release in the culture supernatants was measured by ELISA.

Data shown are the means ± SD of triplicates and are representative of the results of three independent experiments. (b, c, d) **P* <0.01 indicates a statistically significant difference as determined using a T-test. (b, c, d)

Fig. S3: Enhancement of caspase-1 activation in BMDMs by *P. gingivalis* infection under hypoxia is dependent on MyD88.

a,b,d, 200 ng/ml LPS-primed BMDMs derived from wild-type or MyD88-deficient (*Myd88*$^{-/-}$) mice incubated under normoxia (19% O_2) or hypoxia (0.1% O_2) for 12 hours were infected with *P. gingivalis* KDP117 under normoxia or hypoxia. 5 μM nigericin was added to the 200 ng LPS EB-primed BMDMs as control to activate the NLRP3 inflammasome. BHI medium was added as a negative control for inflammasome activation. The cell lysates and culture supernatants were harvested at 12 hpi. The procaspase-1, pro-IL-1β, NLRP3, ASC, β-actin in the cells, subunits of the active-form of active-caspase-1 and mature IL-1β in the culture supernatants were analyzed by immunoblot. IL-1β and IL-6 release in the culture supernatants were measured by ELISA.

c, 200 ng/ml LPS-primed BMDMs were incubated under normoxia (19% O_2), or hypoxia (0.1% O_2) for 12 hours and 5 μM nigericin was added to the BMDMs after

incubation under normoxia or hypoxia for 30 minutes. IL-1β release in the culture supernatants was measured by ELISA.

Blots are representative of the results of three independent experiments. (a,d) Data shown are the means ± SD of triplicates and represent the results of three independent experiments. (b,c) ∗∗P <0.01 indicates a statistically significant difference as determined using a T-test. (b,c)

Fig. S4: HIF-1α expression level is regulated by TRIF under normoxia and *P. gingivalis* infection does not give effect to HIF-1α stability.

a, b, 200 ng/ml LPS-primed BMDMs from wild-type or *Trif*$^{-/-}$ mice were incubated under hypoxia (19% O_2). Cells were harvested to extract RNA or fixed at 8 hours post incubation.

c, LPS-primed BMDMs (200 ng/ml) from wild-type or *Trif*$^{-/-}$ mice incubated under normoxia (21%O_2) or hypoxia (0.1% O_2) were infected with *P. gingivalis* KDP117. Cells were harvested at 4, 8, or 12 hpi.

a, Real-time PCR analysis for expression of HIF-1α mRNA.

b, Immunostaining with Cy3-labeled anti-HIF-1α antibody (orange) and 4_,6-diamidino-2-phenylindole (DAPI, blue) for visualizing the cell nuclei. The merged images are shown. Bar, 20 μm. Staining images are representative of the results of three independent experiments.

c, HIF-1α and β-actin in the cells were analyzed by immunoblot.

Blots and stained images are representative of three independent experiments. (b) Data shown are the means ± SD of triplicates and represent the results of three independent

experiments. (a) ∗$P < 0.05$ indicates a statistically significant difference as determined using a T-test. (a)

Fig. S5: HIF-1α inhibition suppresses the enhanced inflammasome activation by *P. gingivalis* infection under hypoxia.

200 ng/ml LPS-primed BMDMs from wild-type mice were incubated under normoxia (19% O_2), or hypoxia (0.1% O_2) for 12 hours with DMSO or KC7F2, and infected with *P. gingivalis* KDP117 under normoxia or hypoxia. Cell lysates and culture supernatants were harvested at 12 hpi. a, Immunoblot analysis for procaspase-1, pro-IL-1β, and β-actin in the cells and subunits of the active form of active-caspase-1 and mature IL-1β in the culture supernatants. b, ELISA analysis for IL-1β release in the culture supernatants. Blots are representative of three independent experiments. (a) Data are

shown as mean ± SD of triplicates and represent three independent experiments. (b) ∗∗P <0.01 indicates a statistically significant difference using one-way ANOVA with Tukey's test. (b)

Fig. S6: *P. gingivalis* but not *P. intermedia* infection, induced progression of EAE.

Wild-type mice were orally infected with *P. gingivalis* ATCC33277 or *P. intermedia* ATCC25611, or administered carboxymethyl cellulose (CMC) orally on days 0, 2, 4, 6, 8, and 10. Development of EAE in the mice was induced by MOG35-55 and Pertussis toxin. The clinical scores were then monitored for 15 days after the induction. Spleens were collected on day 15 for cytokine analysis. (n=4 mice per group).

a, Schematic of the EAE model infected with *P. gingivalis* or *P. intermedia*. b, Clinical score. c, ELISA analysis for IL-1β release in the spleens. Data shown are the means ±

SD of triplicates and are representative of three independent experiments. (c) $**P < 0.01$ indicates a statistically significant difference as determined by one-way ANOVA with Tukey’s test. (c)
