# Supplementary material for: Hypoxia drives progression of multiple sclerosis by enhancing the inflammasome activation in macrophages with Porphyromonas gingivalis infection
Source: Cell Death Discov. 2025 Jun 10;11:271. doi: 10.1038/s41420-025-02548-z (PMC12152135; doi:10.1038/s41420-025-02548-z)

Source Fig.1b

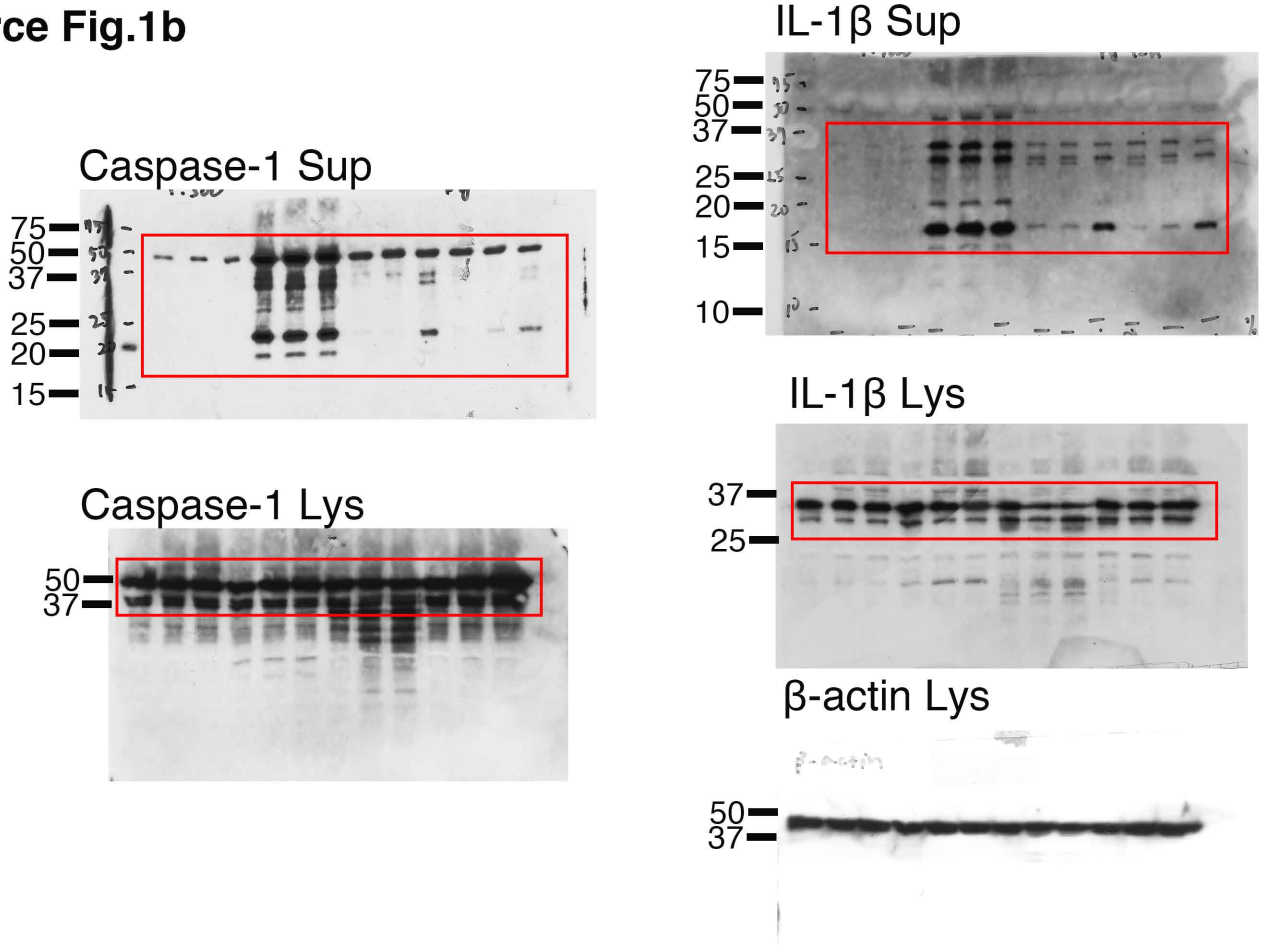

Source Fig. 1d

NLRP3 Lys

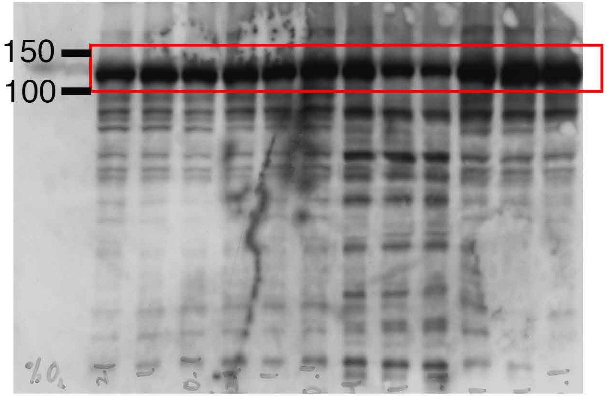

ASC Lys

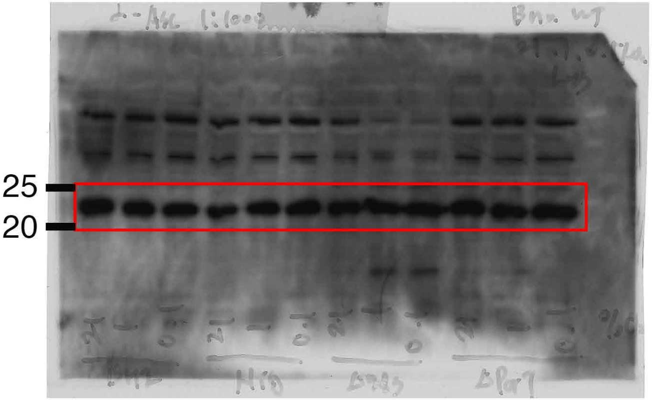

β-actin Lys

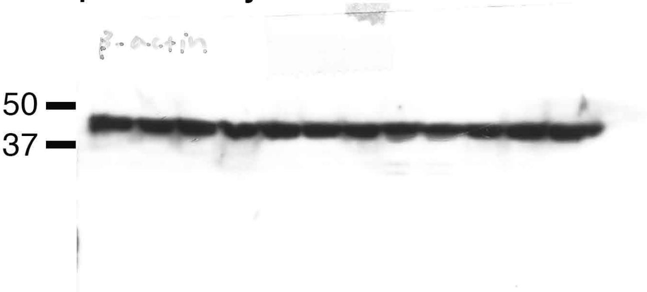

Source Fig. 1f

Caspase-1 Sup

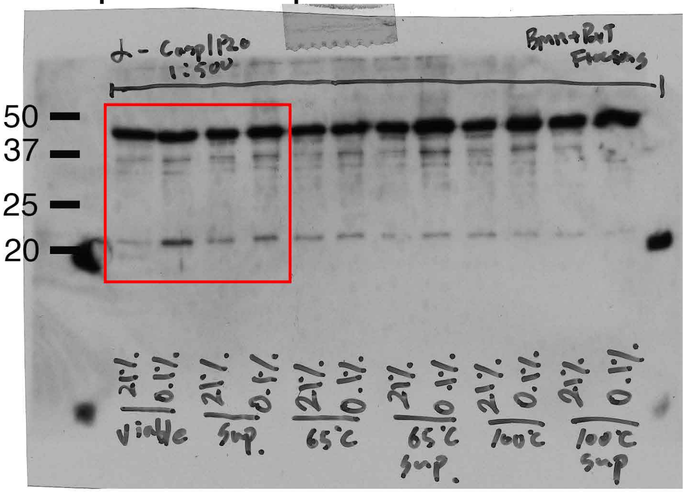

IL-1β Sup

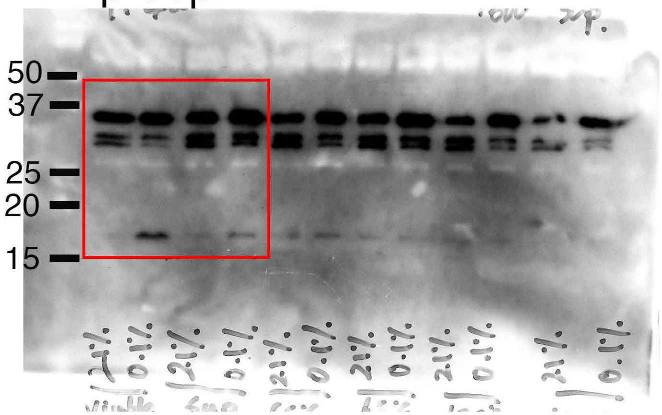

Caspase-1 Lys

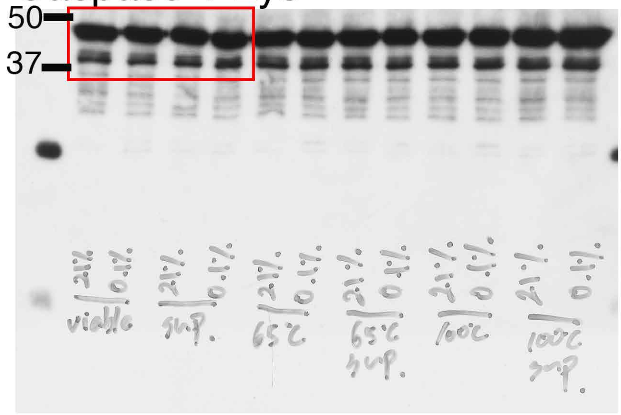

IL-1β Lys

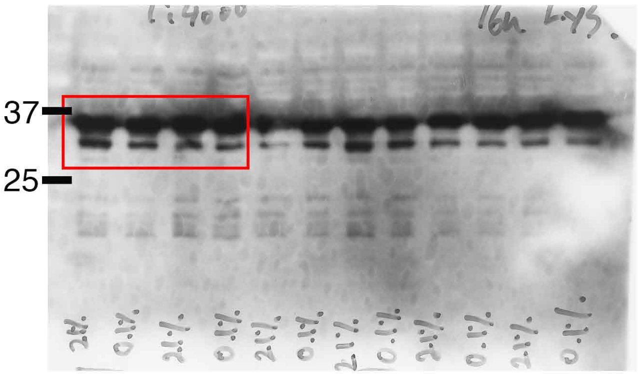

β-actin Lys

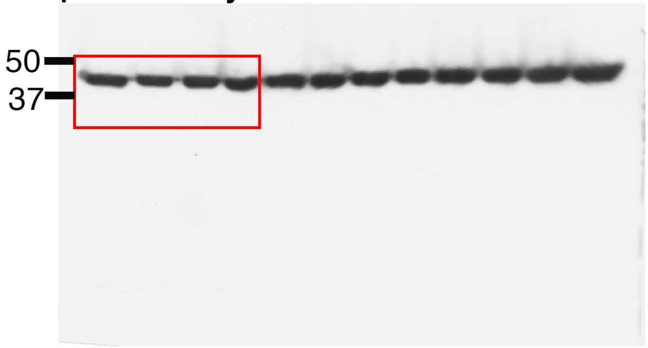

*Actually we used only lane1-4

Source Fig. 2a

Caspase-1 Sup

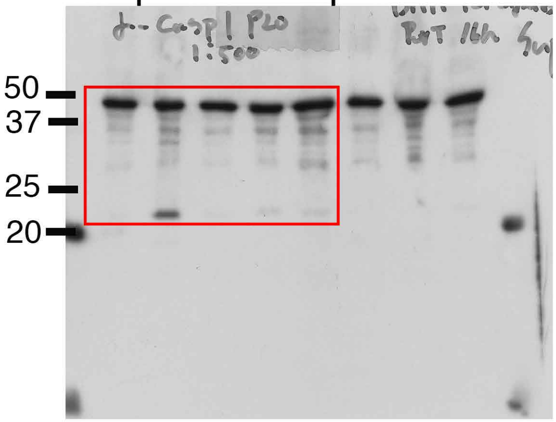

IL-1β Sup

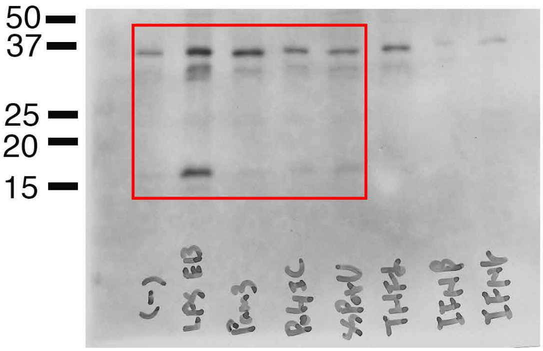

Caspase-1 Lys

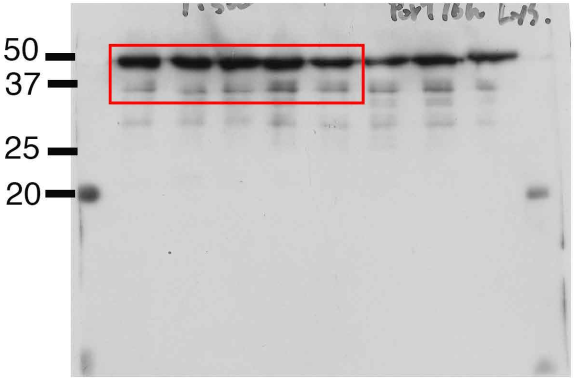

IL-1β Lys

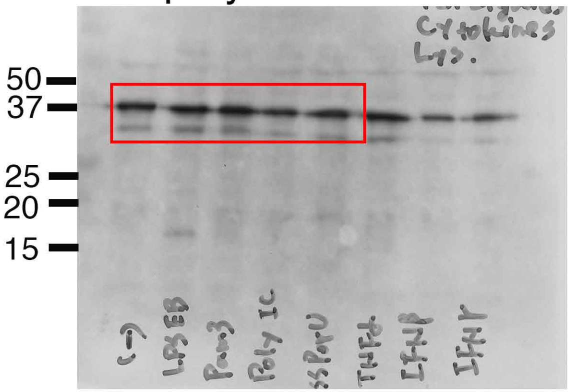

β-actin Lys

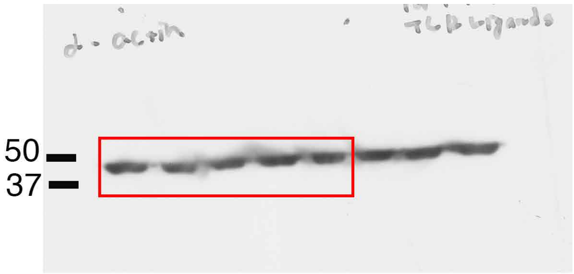

*Actually we used only lane1-5

Source Fig. 2d

NLRP3 Lys

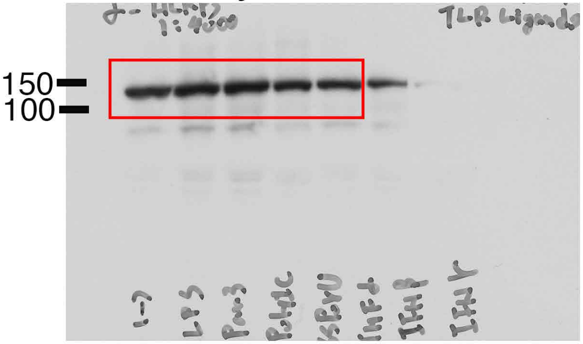

β-actin Lys

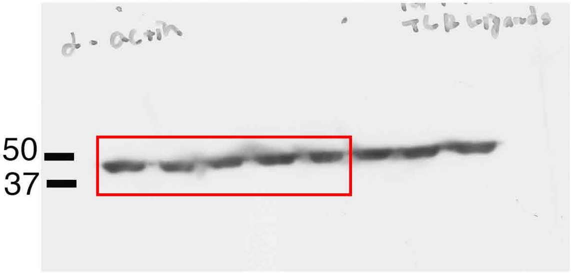

ASC Lys

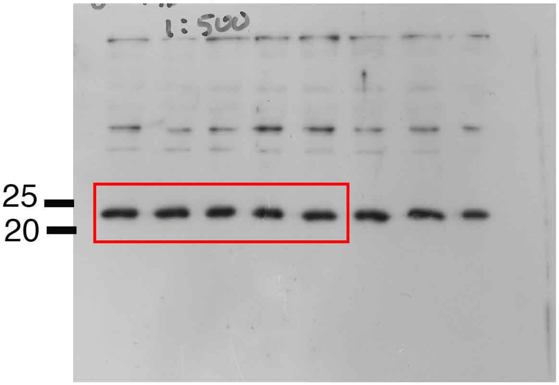

*Actually we used only lane1-5

Source Fig. 2e

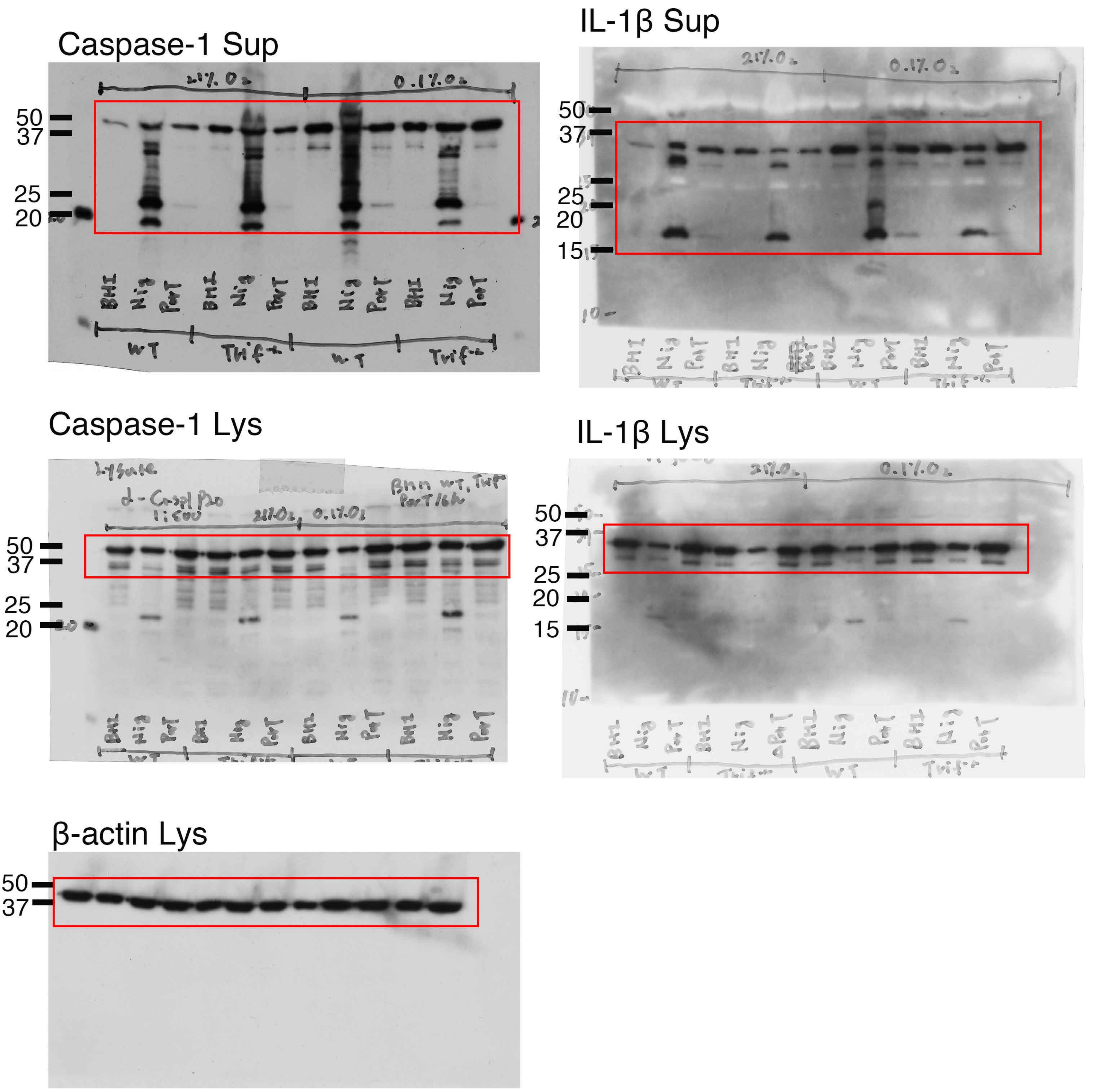

Source Fig. 2h

NLRP3 Lys

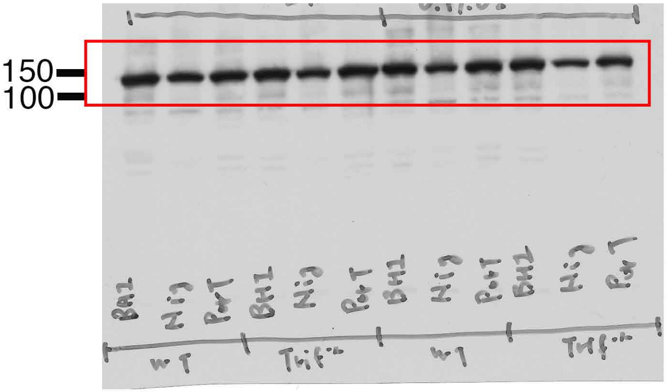

ASC Lys

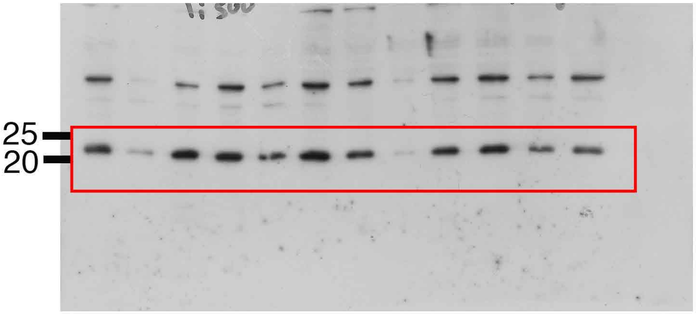

β-actin Lys

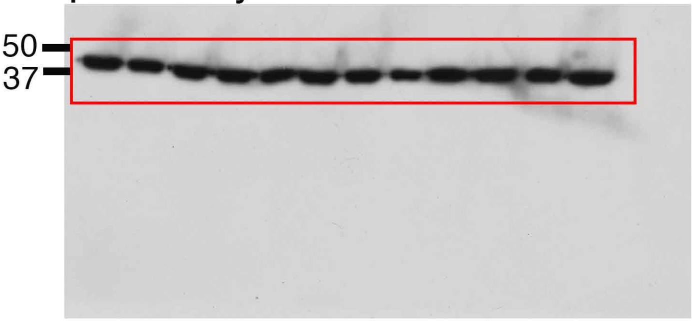

Source Fig. 3e

HIF-1α Lys

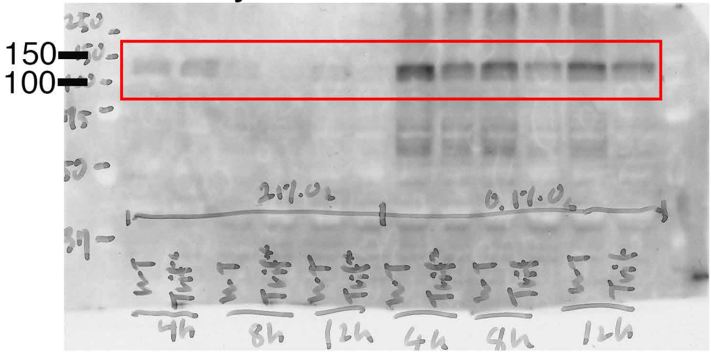

β-actin Lys

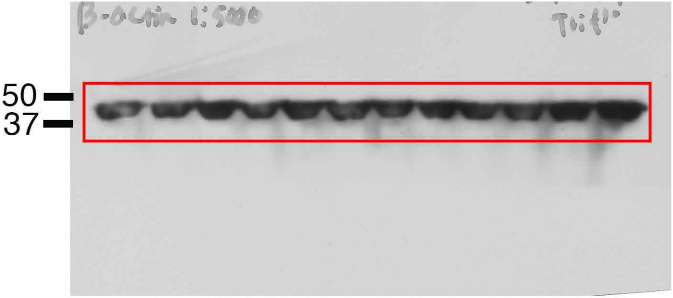

Source Fig. 4a

Caspase-1 Sup

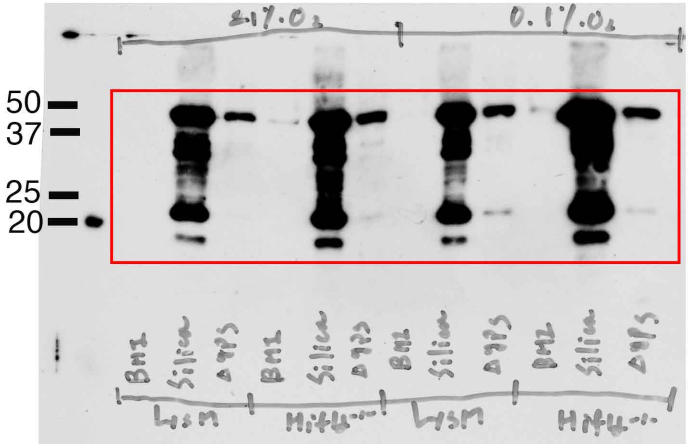

IL-1β Sup

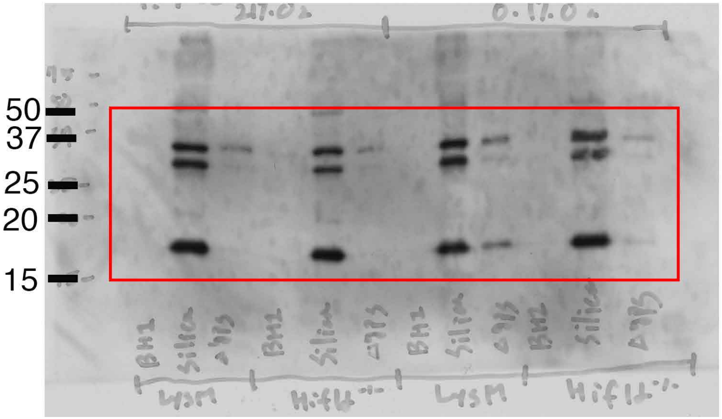

Caspase-1 Lys

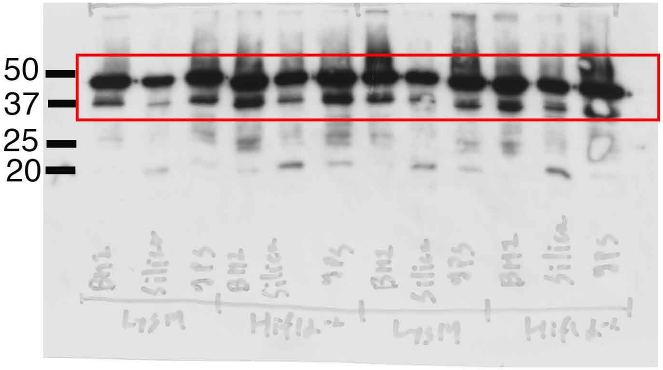

IL-1β Lys

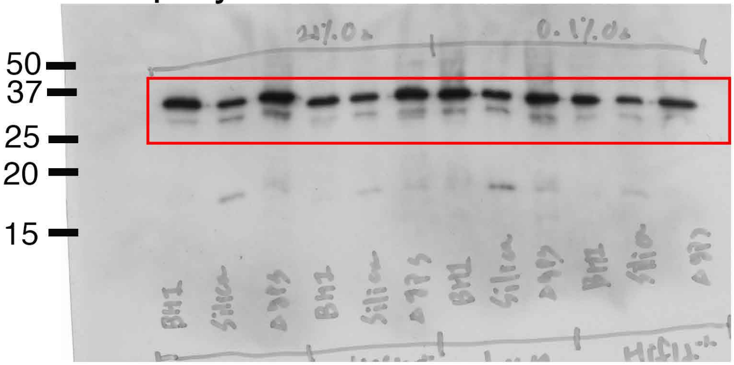

β-actin Lys

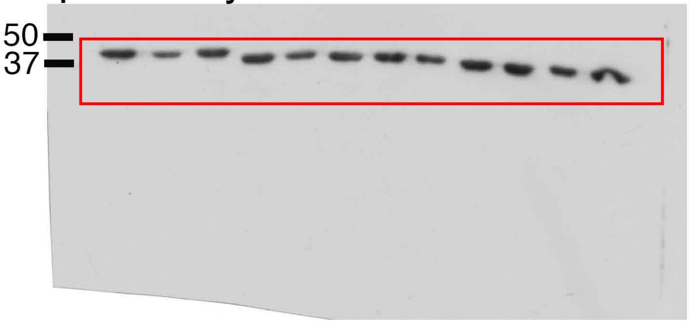

Source Fig. 4 d

NLRP3 Lys

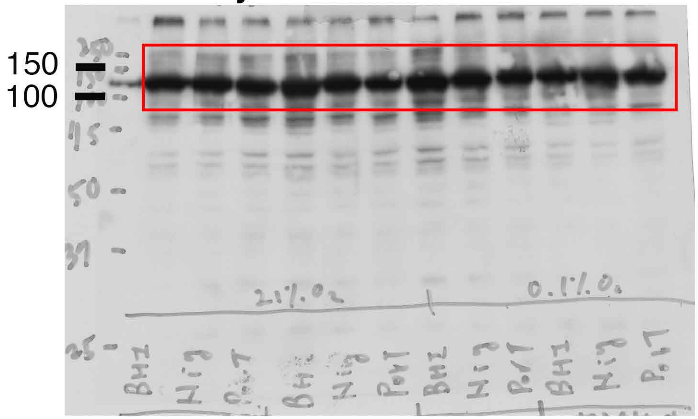

ASC Lys

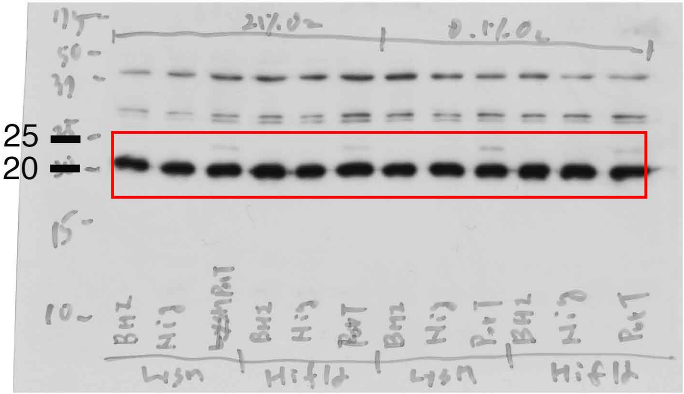

β-actin Lys

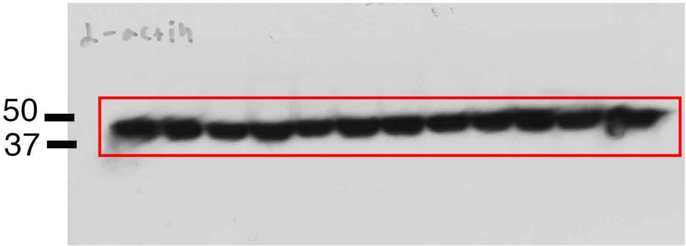

Source Fig. 5d

Caspase-1 P10 in spleen

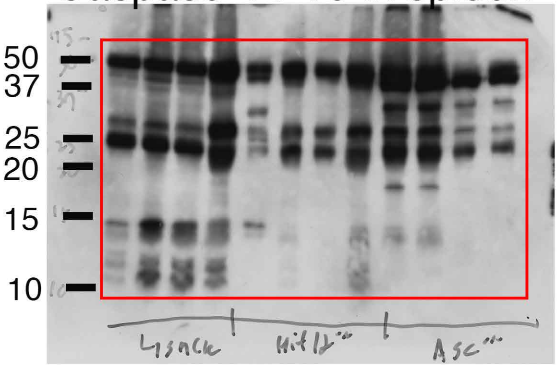

β-actin in spleen

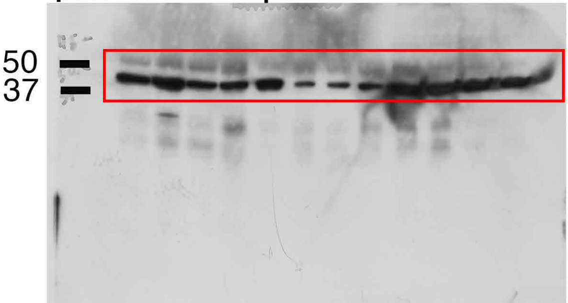

Source Sup Fig. 2a

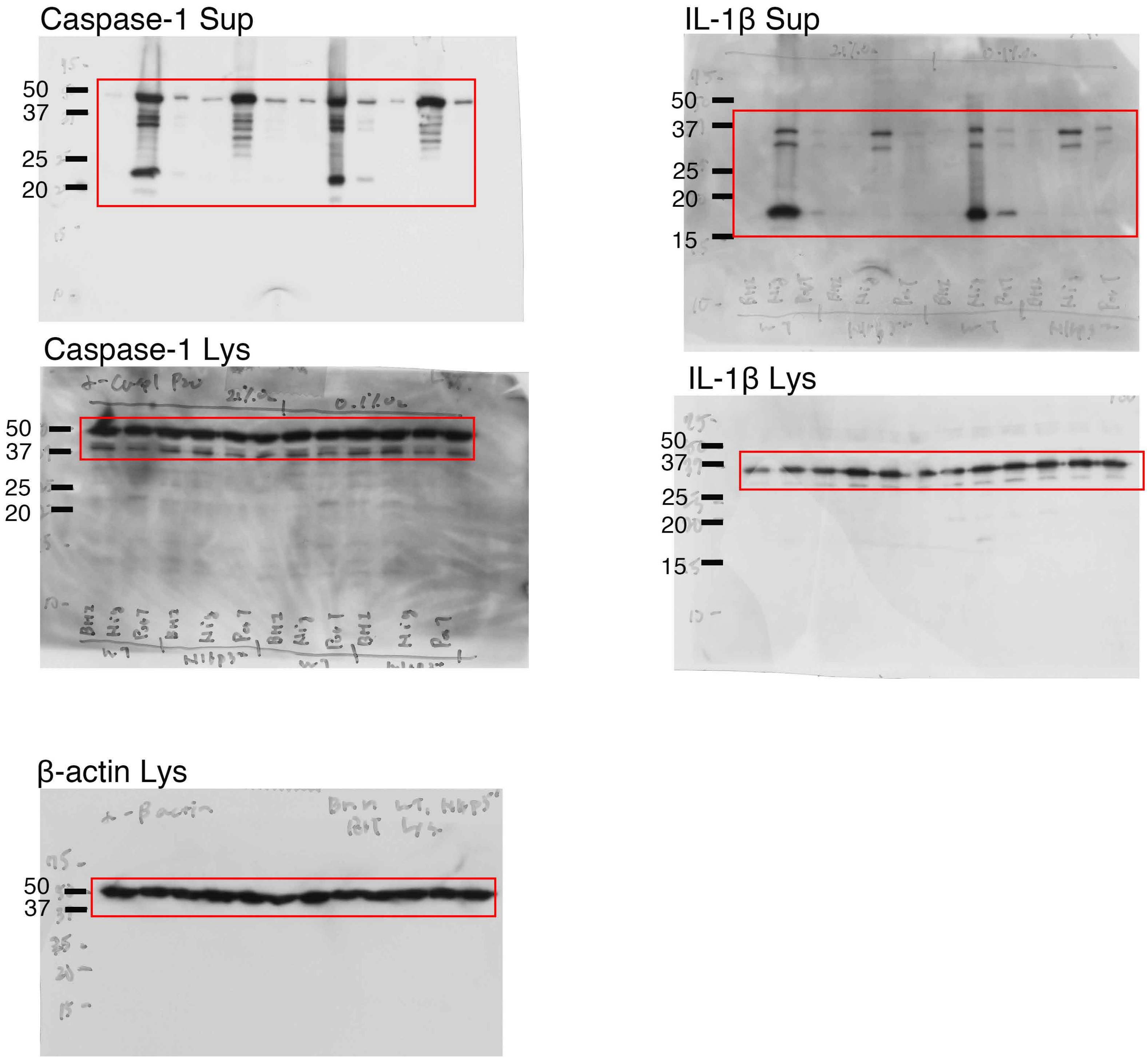

Source Sup Fig. 3a

Caspase-1 Sup

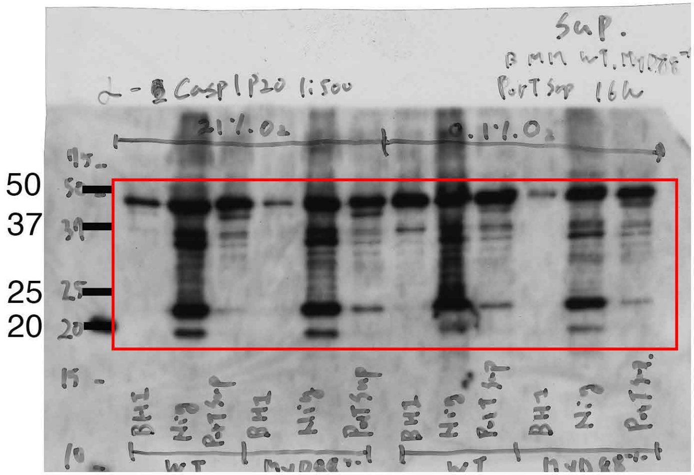

IL-1β Sup

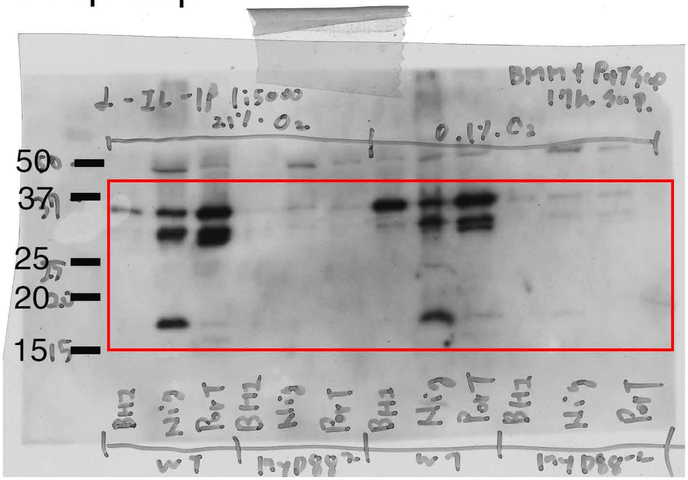

Caspase-1 Lys

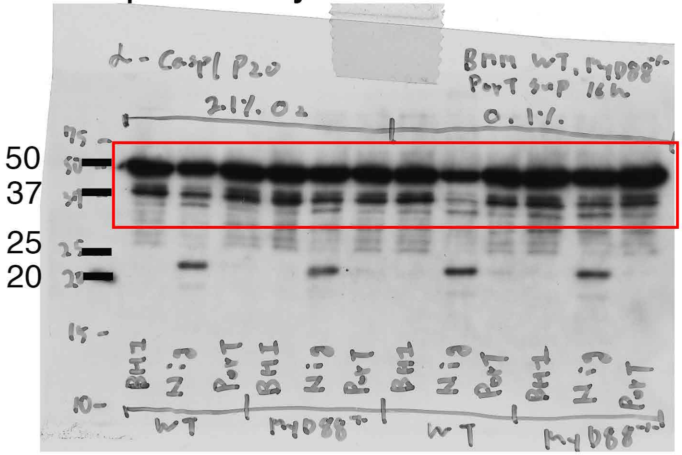

IL-1β Lys

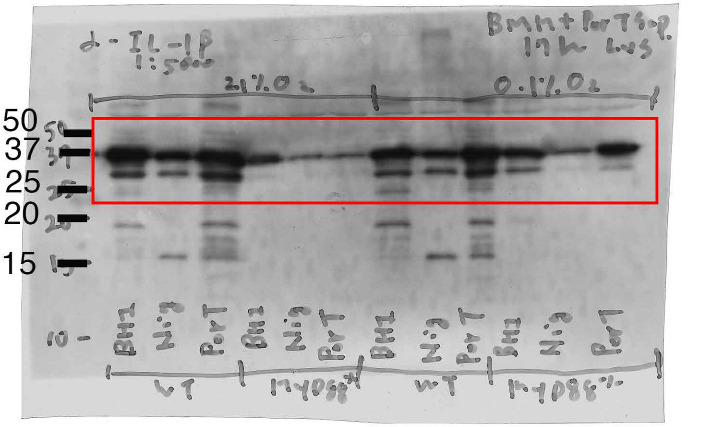

β-actin Lys

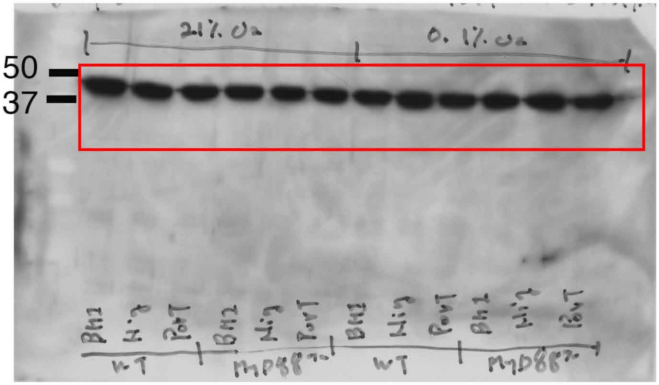

Source Sup Fig. 3d

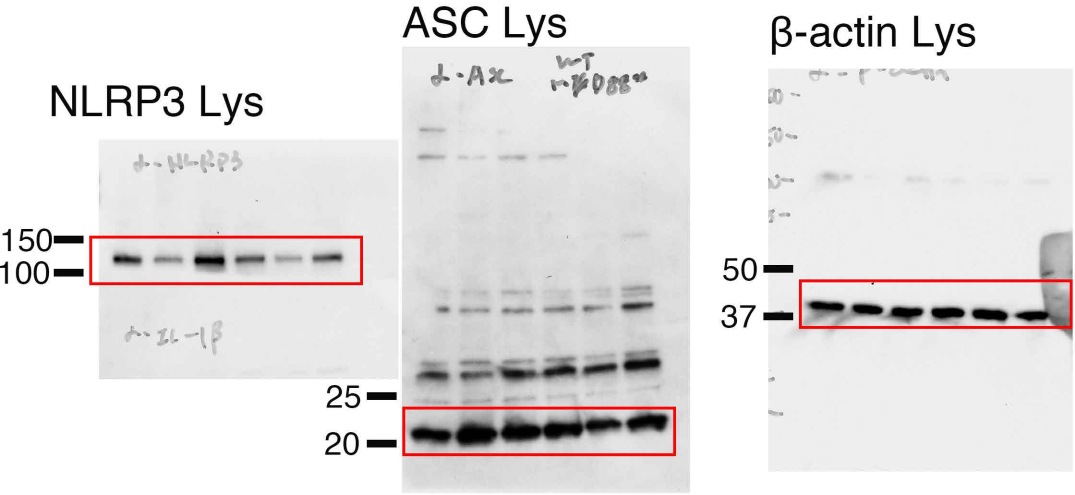

Source Sup Fig. 4c

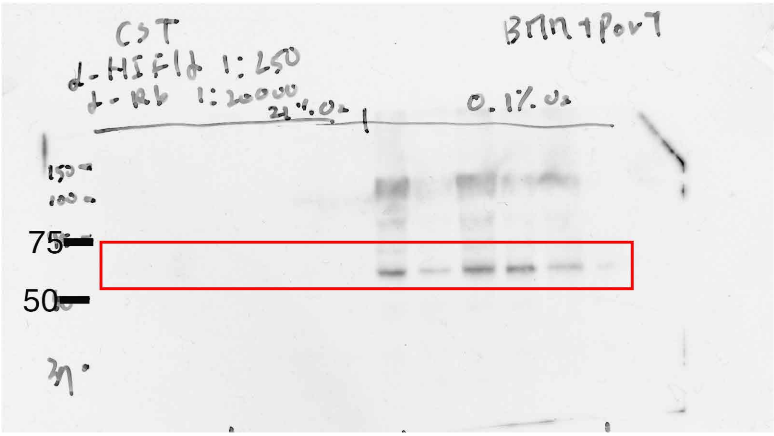

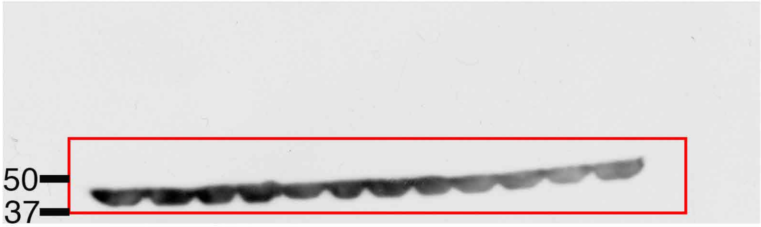

Source Sup Fig. 5a

Caspase-1 Sup

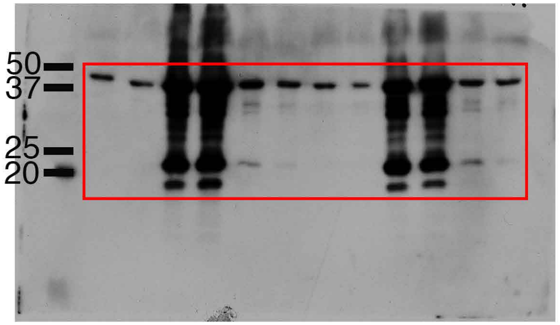

IL-1β Sup

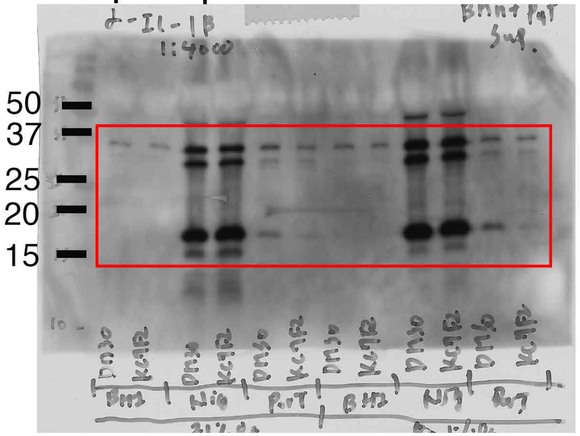

β-actin Lys

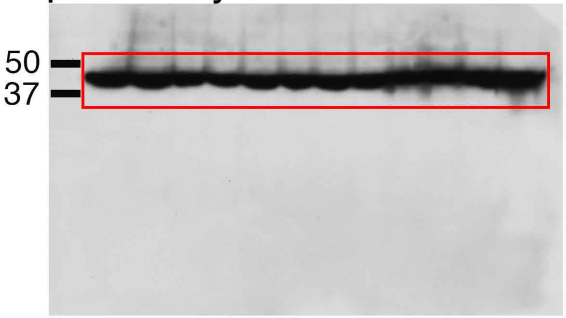

Caspase-1 Lys

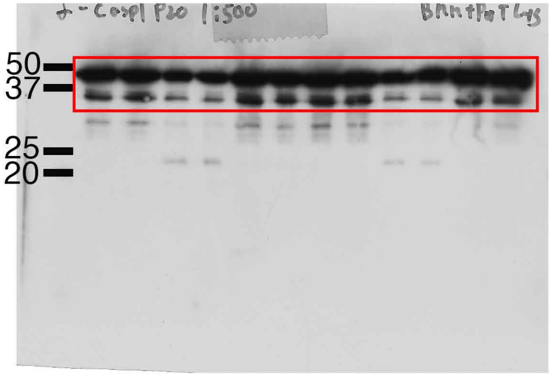

IL-1β Lys

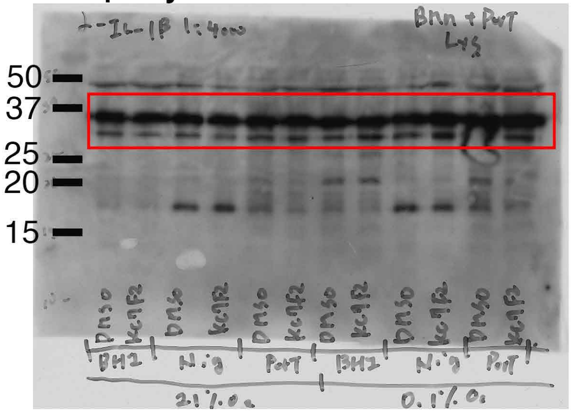

Supplement: Supplementary file 2 — Uncropped images [file 41420_2025_2548_MOESM2_ESM.pdf]
