# Supplementary material for: Hypoxia drives progression of multiple sclerosis by enhancing the inflammasome activation in macrophages with Porphyromonas gingivalis infection
Source: Cell Death Discov. 2025 Jun 10;11:271. doi: 10.1038/s41420-025-02548-z (PMC12152135; doi:10.1038/s41420-025-02548-z)
Supplement: Supplementary file 3 — Table S1 [file 41420_2025_2548_MOESM3_ESM.pdf]

Table S1. Gene expression significantly altered by WT BMDMs
Data are average change relative to TRIF KO group

i. Genes significantly elevated in WT and TRIF KO groups

	GeneSymbol	LogFold Change WT/TRIF KO)
1	Plau	2.96558844
2	Smad3	2.884882765
3	Sox4	2.760856809
4	Myc	2.702625507
5	Cryab	2.672666758
6	Ucp3	2.362160489
7	Prkcb	2.327109572
8	Bcl2	2.144642743
9	Adam8	2.045513872
10	Tgfb2	2.009298917
11	Cpeb1	1.976396451
12	Acot2	1.803603956
13	Zfp36l1	1.709477359
14	Vegfd	1.349734222
15	Camk2d	1.330458399
16	Epas1	1.254976074
17	Tgfb3	1.162025847
18	Adrb2	1.154680503
19	Nol3	1.148825058
20	Cat	1.136082342
21	Cited2	1.113411304
22	Slc2a8	1.109336207

ii. Genes significantly decreased in WT and TRIF KO groups

	GeneSymbol	LogFold Change WT/TRIF KO)
1	Plat	-3.54362209
2	Nos2	-3.372020698
3	Edn1	-3.276074281
4	Kdr	-2.828168921
5	Itpr1	-2.710080476
6	Atp1b1	-2.6203131
7	Pgf	-2.538633195
8	Cd24a	-2.219635356
9	Sod3	-1.958498586
10	Dpp4	-1.919055604
11	Il18	-1.900327526
12	Cav1	-1.793423875
13	Hif1a	-1.732731228
14	Flt1	-1.723125367
15	Eng	-1.635332039
16	Prkce	-1.576388766
17	Fosl2	-1.548508768
18	Loxl2	-1.467029057
19	Mmp2	-1.423984593
20	Casp1	-1.403721635
21	Cd38	-1.255330868
22	Vegfc	-1.158430388
